# Coupled carbonate-shale thallium isotopes reveal the evolution of deep-marine anoxia during the end-Permian mass extinction

**DOI:** 10.1126/sciadv.aef5669

**Published:** 2026-10-28

**Authors:** Susannah C. Herz, Menghan Li, Andrew A. Siciliano, Chadlin M. Ostrander, Sune G. Nielsen, Tapan Mukerji, Stephen E. Grasby, Demir Altiner, Daniel J. Lehrmann, David P. G. Bond, Meiyi Yu, Diego P. Fernandez, Christopher R. Anderson, Jonathan L. Payne, Yanan Shen

**Affiliations:** ^1^ Department of Earth and Planetary Sciences, Stanford University, Stanford, CA 94305, USA.; ^2^ SKL Lunar and Planetary Sciences, Macau University of Science and Technology, Taipa 999078, China.; ^3^ School of Earth and Space Sciences, University of Science and Technology of China, Hefei 230026, China.; ^4^ Department of Geology and Geophysics, University of Utah, Salt Lake City, UT 84112, USA.; ^5^ Department of Geology and Geophysics, Woods Hole Oceanographic Institution, Woods Hole, MA 02543, USA.; ^6^ Centre de Recherches Pétrographiques et Géochimiques, CNRS, Université de Lorraine, Nancy 54501, France.; ^7^ Geological Survey of Canada, Calgary, Calgary, AB T2L 2A7, Canada.; ^8^ Department of Geological Engineering, Middle East Technical University, Ankara 06531, Turkey.; ^9^ Department of Earth and Environmental Geosciences, Trinity University, San Antonio, TX 78212, USA.; ^10^ School of Environmental and Life Sciences, University of Hull, Hull HU6 7RX, UK.; ^11^ Department of Resources and Environmental Engineering, Guizhou University, Guizhou 550003, China.

## Abstract

Ocean anoxia played a key role in the end-Permian mass extinction, the most severe biotic crisis in Earth’s history, yet reconstructions of oxygenation through this event have lacked a focus on deep-water oxygen loss. Thallium isotopes, which track deep-ocean oxygenation via manganese oxide burial, offer a potential solution but have only been applied to siliciclastic sections, limiting spatial and temporal coverage. Here, we present a combined carbonate-shale Tl isotope record, spanning four geographically distant regions and providing a near-continuous record of global-scale redox change during the extinction interval. Both lithologies record deep-marine anoxia beginning 1 million years before the extinction, demonstrating that carbonate- and shale-hosted Tl isotopes preserve consistent redox signals. Late Permian oxygen depletion likely remained largely constrained to the deep ocean and was distinct from the shelf- and slope-depth euxinia of the Early Triassic that was coupled with climate warming and central to the loss of animal diversity.

## INTRODUCTION

The end-Permian mass extinction (EPME) [∼251.906 million years ago (Ma); (*1*)] was the most catastrophic event of Earth’s past half-billion years, taking an especially large toll on marine animal life (*2*). Extensive changes to Earth’s climate and biogeochemical cycles (*3*, *4*) are thought to be related to the estimated release of ∼200,000 billion tonnes (Gt) of CO_2_ into the atmosphere by the Siberian Traps Large Igneous Province, mostly within an interval of 300 thousand years (kyr) (*5*–*7*). Of these disturbances, the combination of >10°C warming (*8*) and widespread free oxygen (O_2_) loss in the oceans (*9*) has been inferred as the primary cause of marine animal extinctions (*10*).

Expanded marine anoxia across the EPME is well documented in the sedimentary and geochemical records of many regions (*9*, *11*–*20*). Numerous paleoredox proxies—such as framboidal pyrite (*13*), biomarkers for green sulfur bacteria (*11*), uranium isotopes (*14*–*16*, *21*), and molybdenum isotopes (*20*, *22*)—are consistent with an increased presence of anoxic and/or euxinic (anoxic with free hydrogen sulfide) conditions above the ancient seafloor. Despite the multitude of evidence for reduced O_2_ availability, uncertainties remain about the location and timing of global marine anoxia during the EPME. The latest mass-balance models for U, the most commonly used global paleoredox proxy, predict that anoxic waters covered 10 to 20% of the global seafloor and were largely restricted to the Early Triassic (*23*, *24*). Interpretations of the U isotope record also suggest that this deoxygenation primarily occurred on continental shelf and slope environments (*15*). However, these findings are incompatible with evidence for widespread deep-ocean deoxygenation (*17*), perhaps reflecting the U isotope proxy’s limited sensitivity to deep-marine environments, where low-productivity conditions could limit U(VI) reduction (*25*). Paleoenvironmental reconstruction of the EPME has lacked a proxy that captures high-resolution global redox histories and is sensitive to O_2_ availability in deep-water environments, limiting our ability to determine the true magnitude, duration, and locus of seafloor anoxia during the EPME.

Thallium (Tl) isotopes have emerged as a promising tool for unveiling detailed redox histories because they are particularly sensitive to the extent of oxygenated seafloor, responding directly to the burial of manganese oxides formed on the deep seafloor (Fig. 1). Manganese responds earlier to changes in O_2_ availability than U and Mo, as dictated by the redox potentials (Eh) of the associated reactions, suggesting that Tl isotopes, which are fractionated by scavenging onto Mn oxides, may be more sensitive to weakly reducing conditions than other global redox proxies (*26*). Furthermore, with a modern seawater residence time of 18.5 kyr, Tl provides higher temporal resolution than alternative global redox proxies such as U and Mo [with residence times of ∼450 kyr; (*27*, *28*)], capturing variations on timescales as short as millennia (*29*) and potentially submillennia in periods of extreme anoxia (*30*). By contrast, proxies with residence times shorter than the ocean mixing time of ∼1 kyr [e.g., Fe speciation and Ce anomalies; (*19*, *31*)] respond to local conditions, making them harder to use as tracers of global conditions during mass extinction events like the EPME. Assuming that the residence time of Tl remained similar to the modern value across the Permian-Triassic boundary (PTB), Tl isotopes can capture global trends in redox conditions in greater detail than what has been possible previously. For example, Newby *et al.* (*18*) applied the Tl isotope proxy to shale samples from this time interval, identifying signals of prolonged ocean anoxia before the extinction and a short reoxygenation (on the timescale of tens of thousands of years) immediately before the EPME.

**Fig. 1.**
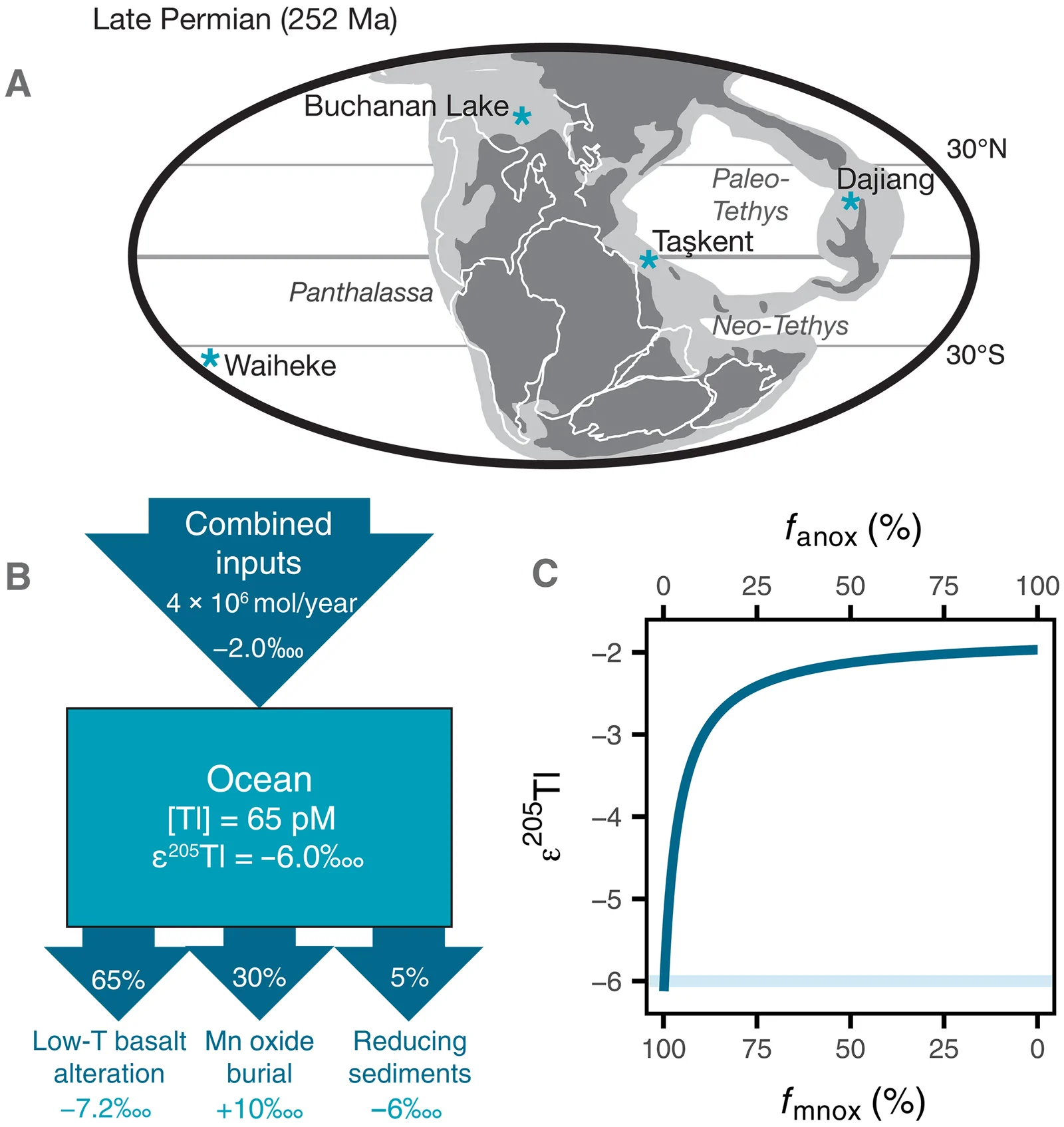
Section locations and thallium isotope box model. (**A**) Paleogeographic map for the Late Permian (252 Ma) showing the locations of the four sections. Reprinted from (*95*), with permission from Elsevier. (**B**) Box model illustrating the inputs and outputs of Tl to and from the ocean according to the modern conventional mass balance. (**C**) Mass-balance-derived ε^205^Tl_sw_ values as a function of *f*_mnox_ (the fraction of the seafloor that is oxygenated enough to support the burial of Mn oxide minerals such as hexagonal birnessite) at steady state. The upper *x* axis shows the complementary fraction, *f*_anox_—the portion of the seafloor too poorly oxygenated for Mn oxide burial (*f*_anox_ = 1 − *f*_mnox_). The light blue line represents the ε^205^Tl value for modern, well-oxygenated seawater.

To date, Tl isotopes have been applied primarily as a paleoredox proxy to low-oxygen siliciclastic sedimentary sequences, which can provide robust archives of seawater Tl isotope compositions (*29*, *32*–*39*). In such sediments, Tl is primarily sequestered from seawater into sulfide minerals (*34*, *40*, *41*), sometimes reaching concentrations as high as a few parts per million (ppm) (*38*). However, deposition of shale beneath anoxic bottom waters is often discontinuous, interrupting proxy records. In carbonates, which are often deposited more continuously and with higher sedimentation rates (*42*, *43*), Tl concentrations are typically on the order of single to tens of parts per billion (ppb) (*44*), which has delayed the use of the Tl isotope proxy in carbonates (*45*). This challenge and others (e.g., potential fractionations during Tl incorporation and diagenesis) have been addressed by recent work on the Great Bahama Bank, where modern and recent carbonates faithfully record seawater Tl isotope values (*44*). Expanding the use of the Tl isotope proxy to ancient carbonates would enable the development of proxy records with greater time resolution and completeness. It would facilitate comparisons among contemporaneous sections of different lithologies without requiring any assumptions about differential fractionation between the records, as is necessary for proxies such as U isotopes (*27*).

Here, we present contemporaneous carbonate and shale Tl isotope records from four globally distributed sections that span the PTB (Fig. 1A). This combined record allows us to assess the applicability of the Tl isotope redox proxy to ancient carbonate rocks. It also provides a higher-resolution redox history of the EPME and captures the loss of oxygenated deep-water environments undetected by alternative isotope systems. We use an inverse modeling approach to estimate the fraction of the seafloor that was sufficiently oxygenated to support Mn oxide burial during this interval, providing improved constraints on the timing, extent, and tempo of deoxygenation that led to the EPME.

### Thallium as a paleoredox proxy

Thallium isotope compositions (ε^205^Tl) represent the relative abundances of the two stable isotopes of Tl, ^203^Tl and ^205^Tl, reported relative to the NIST SRM 997 standard

ε205Tl (‱)=[(Tlsample205/203/TlNIST−SRM−997205/203)−1]×10,000 (1)

There are multiple inputs of Tl to the ocean, including transport of weathered material through rivers, subaerial volcanism, hydrothermal fluids, and mineral aerosols (*46*, *47*). The globally averaged ε^205^Tl value of these inputs is −2‱, indistinguishable from the average bulk upper continental crust (*48*). Modern seawater is comparatively enriched in lighter-mass ^203^Tl and has a globally homogeneous ε^205^Tl value of −6‱ set by isotope fractionation effects imparted by the important marine outputs (*47*). One of these outputs is low-temperature basalt alteration, which preferentially extracts ^203^Tl and causes an average of −1.2‱ difference between seawater and the altered crust (*47*). Reducing sediments are another important Tl output (*49*). Thallium transferred to these sediment types (and held primarily in sulfide minerals like pyrite) is unfractionated, resulting in ε^205^Tl values indistinguishable from overlying waters (*41*, *49*).

The final important output of marine Tl is the precipitation and burial of manganese oxide minerals on the seafloor. This process preferentially removes heavier-mass ^205^Tl, leading to ε^205^Tl differences from seawater of up to +20‱ when the mineral with the strongest ^205^Tl affinity (hexagonal birnessite) is present in large quantities (*50*–*53*). Manganese oxides become unstable in sediments when oxygen concentrations decrease to dysoxic levels (*54*, *55*), making ε^205^Tl_sw_ sensitive to changes in global bottom-water redox conditions. A seawater ε^205^Tl value that is lower than the input of −2‱ indicates the operation of the Mn oxide output (*47*). For this situation to occur, not only must oxygen be present at the sediment-water interface in at least some regions of the ocean, but these areas must also be oxygenated consistently to avoid reductive dissolution of Mn oxides (*56*). Furthermore, these Mn oxides must be buried in the sediments long-term to result in Tl isotope fractionation (*34*). Manganese oxides buried in deep-water environments result in much larger Tl isotope fractionations than those buried nearshore (*50*). For this reason, the Tl isotope proxy is primarily considered a proxy for deep-ocean oxygenation (*34*, *39*). An increase in ε^205^Tl_sw_ toward the −2‱ global inputs value implies attenuation of global Mn oxide burial fluxes, which suggests reduced extent of oxygenated seafloor. The relationship between ε^205^Tl and the area of the seafloor that supports Mn oxide burial (*f*_mnox_) is nonlinear, with Tl isotopes being the most sensitive to 80 to 100% sufficiently oxygenated seafloor, below which ε^205^Tl values within error of the crustal baseline of −2‱ are reached.

## RESULTS

### Thallium isotopes

We measured Tl isotope ratios for two carbonate (Dajiang, eastern Tethys and Taşkent, western Tethys) and two shale-dominated (Buchanan Lake, NW Pangea and Waiheke, abyssal Panthalassa) PTB sections. The carbonate and shale ε^205^Tl records display broadly similar trends before and after the extinction (Fig. 2). In the Dajiang carbonate section, ε^205^Tl exhibits a gradual decline from 0.2 to −6.2‱ in the lead-up to the extinction horizon, reaching the lowest value immediately before the extinction. The ε^205^Tl values shift to −1.4‱ across the extinction boundary, followed by another less extreme negative excursion to −3.7‱ in the Lower Triassic. The Taşkent carbonate section also begins close to 0‱ and declines to −5.3‱ shortly before the extinction. Similarly to Dajiang, ε^205^Tl shifts toward higher values across the extinction boundary, peaking at −0.6‱, and then undergoes a brief negative excursion to −2.7‱, remaining at ε^205^Tl values near −1 to −2‱ for the rest of the measured interval in the Lower Triassic. In the Buchanan Lake shale section, ε^205^Tl values begin close to 0‱ and gradually decrease toward −4.8‱ just before the EPME. This shift is interrupted by what appears to be a brief negative excursion to −4.2‱ midway through the pre-extinction interval. Across the PTB, there is an excursion to ε^205^Tl as high as −0.8‱ before an eventual stabilization at values near −2‱ farther into the Induan substage. In mudstones from the Waiheke section, the ε^205^Tl values are comparatively higher pre-extinction, remaining between −1.8 and +1.2‱, before an abrupt shift to −2.6‱ at the end of the Changhsingian. A minimum of −4.1‱ is reached directly after the extinction within the same bed. The Waiheke record terminates here due to insufficient Tl concentrations for isotopic analysis above this level.

**Fig. 2.**
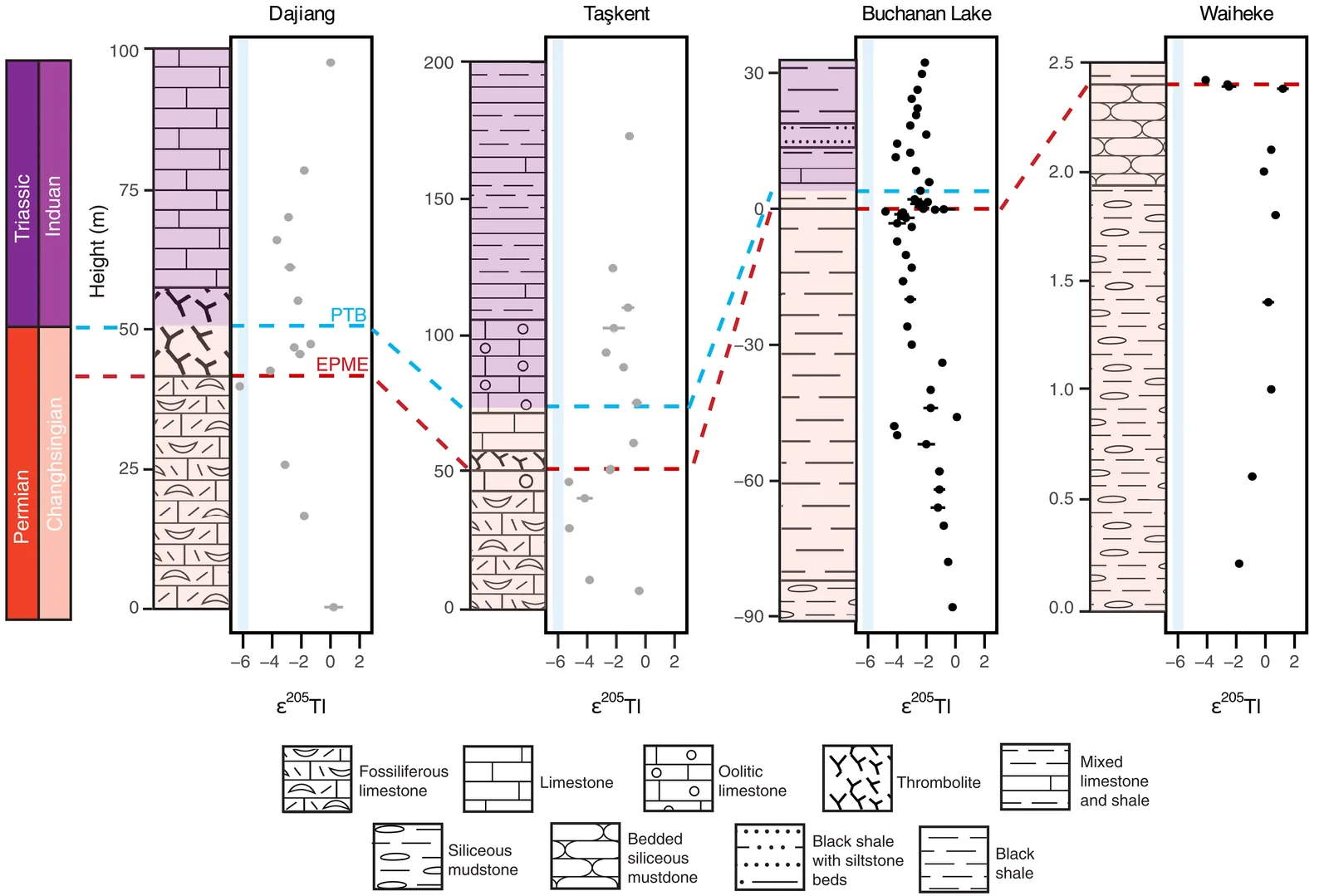
ε^205^Tl measurements from the carbonate sections (Dajiang and Taşkent) and the shale-dominated sections (Buchanan Lake and Waiheke). The light blue lines represent the ε^205^Tl of modern, well-oxygenated seawater. The red dashed line marks the EPME (∼251.906 Ma), and the blue dashed line marks the PTB (∼251.867 Ma) (*1*). Error bars represent ±2 SD; where not visible, they are smaller than the plotted symbols.

### Diagenesis and alteration

Carbonate sediments often experience water-rock exchange and precipitation of cement phases during burial, both of which can cause diagenetic alteration of sedimentary Tl isotope ratios for these Tl-poor sediment types. Therefore, assessing for signs of alteration is crucial for determining whether the ε^205^Tl_carb_ signal reliably reflects seawater values. Elevated Mg/Ca ratios can be a sign of open-system recrystallization or dolomitization and high Mn/Sr ratios may indicate alteration via burial diagenesis (*57*). ε^205^Tl_carb_ has a weak inverse correlation with Mg/Ca in both the Dajiang and Taşkent sections and ε^205^Tl_carb_ shows no significant correlation with Mn/Sr (fig. S1). In addition, ε^205^Tl_carb_ is not strongly correlated with Mn concentration, indicating that sample digestion does not appear to be extracting Tl from Mn oxides in quantities that would affect the overall interpretation. Elevated Mn/Tl ratios may indicate that a larger proportion of Tl in the samples originate from Mn oxides. We plot the average Mn/Tl ratio of hydrogenetic ferromanganese crusts (*50*) alongside Mn and Tl concentrations in our samples, finding that our samples do not follow this trend and that there is only a weak and nonsignificant correlation between Mn and Tl, further suggesting that Mn oxides are not strongly affecting the ε^205^Tl_carb_ signal. Potential influence from detrital components was evaluated by examining relationships between ε^205^Tl_carb_ with Al and Th, neither of which are significantly correlated with Tl isotope compositions. There is a strong inverse correlation between ε^205^Tl_carb_ and Tl concentration in the Dajiang section (ρ = −0.72, *P* = 0.002) and a moderate but not statistically significant inverse correlation in the Taşkent section (ρ = −0.45, *P* = 0.1), which is opposite of what would be expected from Mn oxide contamination and is perhaps related to expanded anoxia, in which ε^205^Tl increases and the size of the dissolved seawater Tl reservoir decreases.

Shales and other siliciclastic-dominated sediments only reflect seawater ε^205^Tl values when deposited in locally reducing conditions. Therefore, constraining the local redox environment is critical for evaluating the preservation of ε^205^Tl signals in siliciclastic sediments. At Buchanan Lake, local anoxia persisted through the Late Permian and into the Early Triassic based on redox sensitive trace-metal enrichments (*12*) and δ^15^N > 9‰ (per mille) paired with high concentrations of lycopane biomarkers, indicative of denitrification and persistently suboxic to anoxic bottom waters (*58*). The Waiheke section is also characterized by progressive development of anoxia through the latest Permian and transient euxinia across the PTB based on Fe speciation (*19*) and redox-sensitive trace-metal enrichments (*17*).

### Modeled oxygenation trends

Redox proxy records can be interpreted qualitatively or semiquantitatively on the basis of measured values alone. However, for a high-resolution record of a system such as Tl, where there is a nonlinear relationship between ε^205^Tl and the amount of deoxygenation present (Fig. 1C), a dynamic, statistical model is needed to determine the best reconstruction and its uncertainties. This approach has the further advantage of addressing the inevitable noise in the data and identifying shared signal across records. Sedimentary Tl isotope records can be quantitatively linked to the output flux of Mn oxide burial (*F*_mnox_) through isotope mass balance. This relationship enables estimation of the fraction of seafloor area sufficiently oxygenated to support the formation and long-term burial of Mn oxide minerals that result in large Tl isotope fractionations (*f*_mnox_; Fig. 1B), namely, hexagonal birnessite (*50*–*53*), thereby revealing large-scale patterns in deep-ocean oxygenation. Although the precise O_2_ concentration required to support this process remains uncertain, observations from multiple marine environments show Mn oxide burial declines rapidly under dysoxic [<2 ml/liter; (*59*)] conditions (*54*, *55*).

We compare our Tl isotope results to those of Newby *et al.* (*18*) and Wang *et al.* (*60*) by aligning all ε^205^Tl records from the Permian-Triassic interval on a common age model (Fig. 3 and fig. S2; see the Supplementary Materials). Newby *et al.* (*18*) measured ε^205^Tl in three siliciclastic-dominated PTB sections (the deep Panthalassa Gujo-Hachiman and Ubara sections in Japan and the western Pangea shelf Opal Creek section in Alberta, Canada). Wang *et al.* (*60*) developed two additional ε^205^Tl records from the shale component of samples from the Meishan section, a shallow-marine carbonate environment, and from the Lengqinggou section, a marine-terrestrial transitional environment, both in South China. The Lengqinggou record was excluded from our analyses because a substantial portion of its ε^205^Tl measurements originate from the Xuanwei Formation, a unit interpreted as a terrestrial, freshwater peat-swamp deposit that is unlikely to record seawater ε^205^Tl values (*61*).

**Fig. 3.**
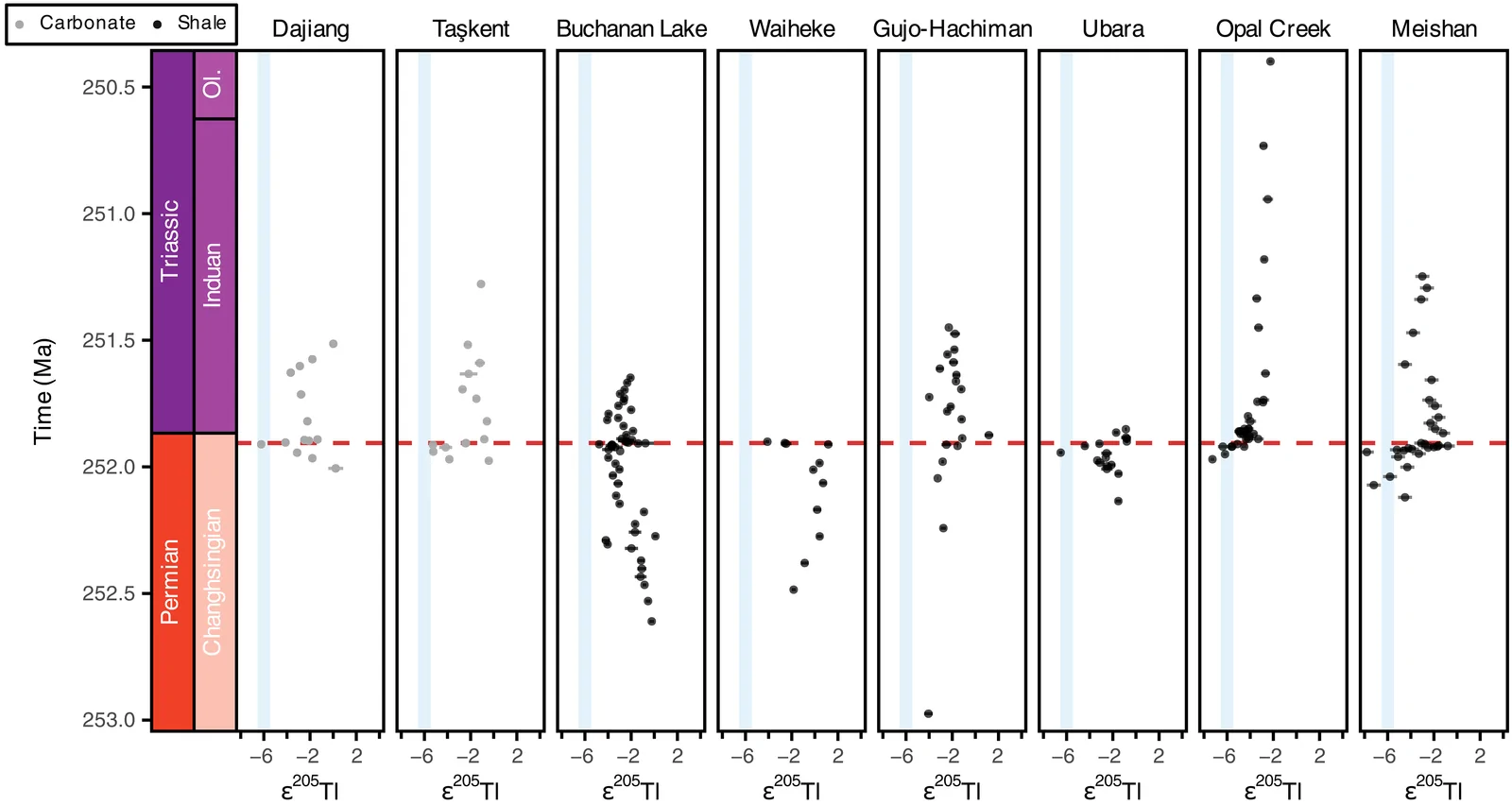
Age correlation of all published Permian-Triassic ε^205^Tl records. See the Supplementary Materials for details on the correlations, including the Dajiang, Taşkent, Buchanan Lake, and Waiheke sections (this study), the Gujo-Hachiman, Ubara, and Opal Creek sections (*18*), and the Meishan section (*60*). Capitanian-age data from Opal Creek are not included due to an unconformity preventing precise age estimates. The light blue lines represent the ε^205^Tl of modern, well-oxygenated seawater. The red dashed line marks the EPME [∼251.906 Ma; (*1*)]. Error bars represent ±2 SD; where not visible, they are smaller than the plotted symbols. Ol., Olenekian.

Using an inverse model, we analyze this data compilation to predict quantitative changes in *f*_mnox_ driven by expanded dysoxic conditions through the extinction interval (see the Supplementary Materials for details on model construction and sensitivity analyses). Although the relationship between ε^205^Tl and *f*_mnox_ can be calculated from the steady-state mass balance (Fig. 1C), this method assumes that each data point represents steady-state conditions, which requires that sufficient time has elapsed, ∼5 residence times (*24*). Our model instead allows for non-steady-state behavior between measurements, a more appropriate assumption for a high-resolution ε^205^Tl record with less than 100 kyr between data points. The model predicts no substantial Mn oxide burial during the ∼1 million years (Myr) before the main extinction event, indicative of extensive seafloor deoxygenation during this time (Fig. 4). The model also reconstructs a sharp increase in the flux of Mn oxide burial immediately before the extinction (maximum *F*_mnox_ of 8%; *f*_mnox_ of 90%). Notably, the 16th to 84th error interval spans all possible values of *f*_mnox_ (i.e., 0 to 100%) during this feature. These large uncertainties partly reflect the limited sensitivity of the Tl isotope proxy in the 0 to 80% *f*_mnox_ range (Fig. 1C), where ε^205^Tl values fall within analytical error of the −2‱ crustal baseline.

**Fig. 4.**
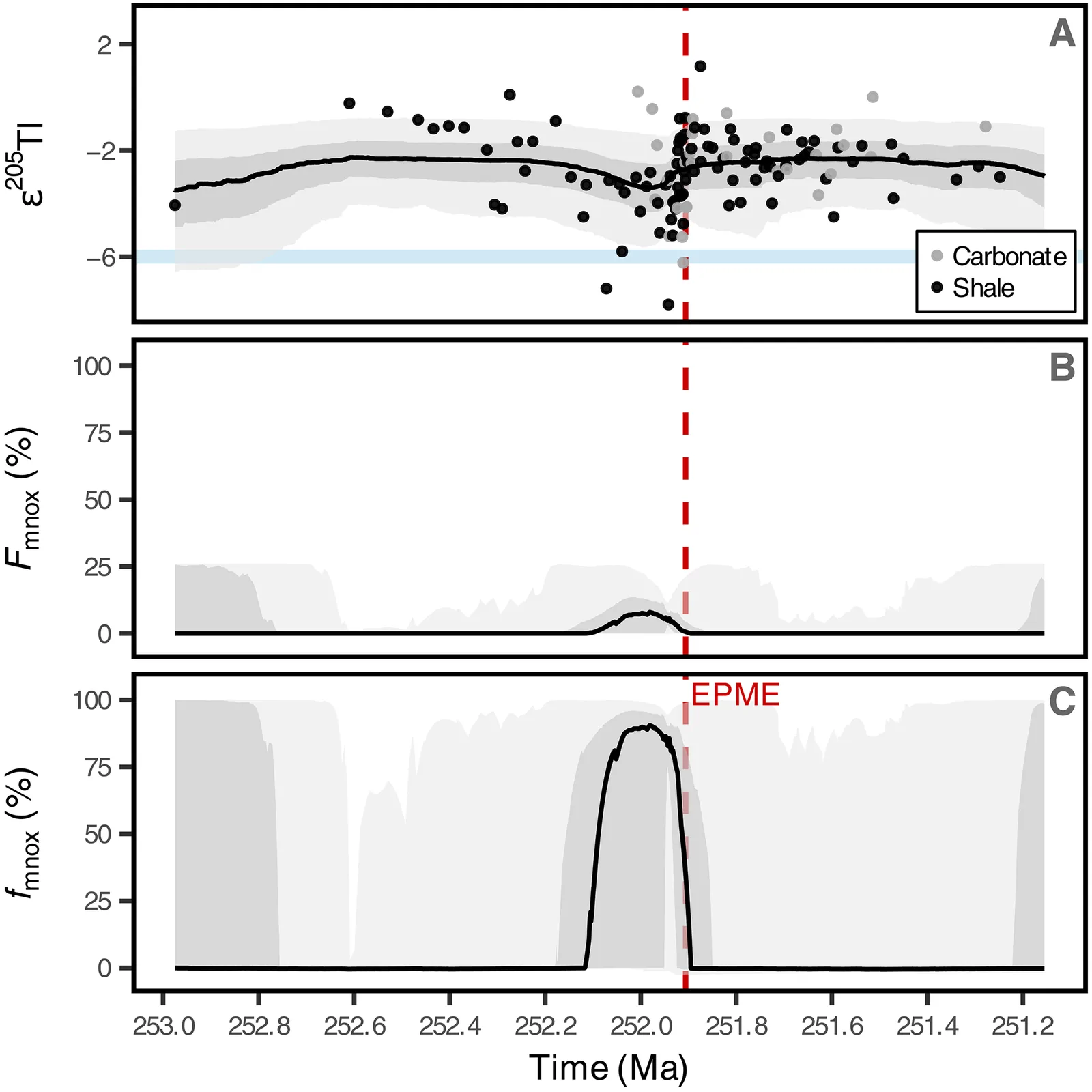
Inverse modeling results. (**A**) Fit of the MCMC simulations to the ε^205^Tl data from the Dajiang, Taşkent, Buchanan Lake, Gujo-Hachiman, and Meishan sections, (**B**) the percent of Tl inputs going toward Mn oxide burial (*F*_mnox_), and (**C**) the predicted fraction of seafloor area sufficiently oxygenated to support Mn oxide formation in the form of hexagonal birnessite (*f*_mnox_) over the study interval. Black lines represent the median model output, light gray shading indicates the 16th to 84th percentile interval, and dark gray shading indicates the 33rd to 66th percentile interval. The light blue line indicates the ε^205^Tl value of modern, well-oxygenated seawater. The red dashed line marks the EPME [∼251.906 Ma; (*1*)].

## DISCUSSION

### Assessing Tl isotope proxy preservation

The major ε^205^Tl features initially observed in PTB records from equatorial and mid-latitude Panthalassa are geographically reproducible in the eastern and western Paleo-Tethys Ocean, at high and low latitudes in Panthalassa, and between carbonates and shales (Figs. 2, 3, and 5 and fig. S2). The samples used in this study are unlikely to have been affected by basinal restriction based on the similarity of the Tl isotope records among sites and the agreement of other geochemical proxies (e.g., δ^13^C, ^87^Sr/^86^Sr, and δ^238^U) with global trends (*15*, *17*, *62*, *63*). Paleoenvironmental interpretations of these sites also suggest that they maintained exchange with the global ocean (*64*–*68*). The two carbonate ε^205^Tl records are in excellent agreement with each other (Fig. 5A), despite the higher potential for postdepositional modification of Tl isotopes in carbonates relative to shales due to the much lower Tl concentrations in carbonates.

**Fig. 5.**
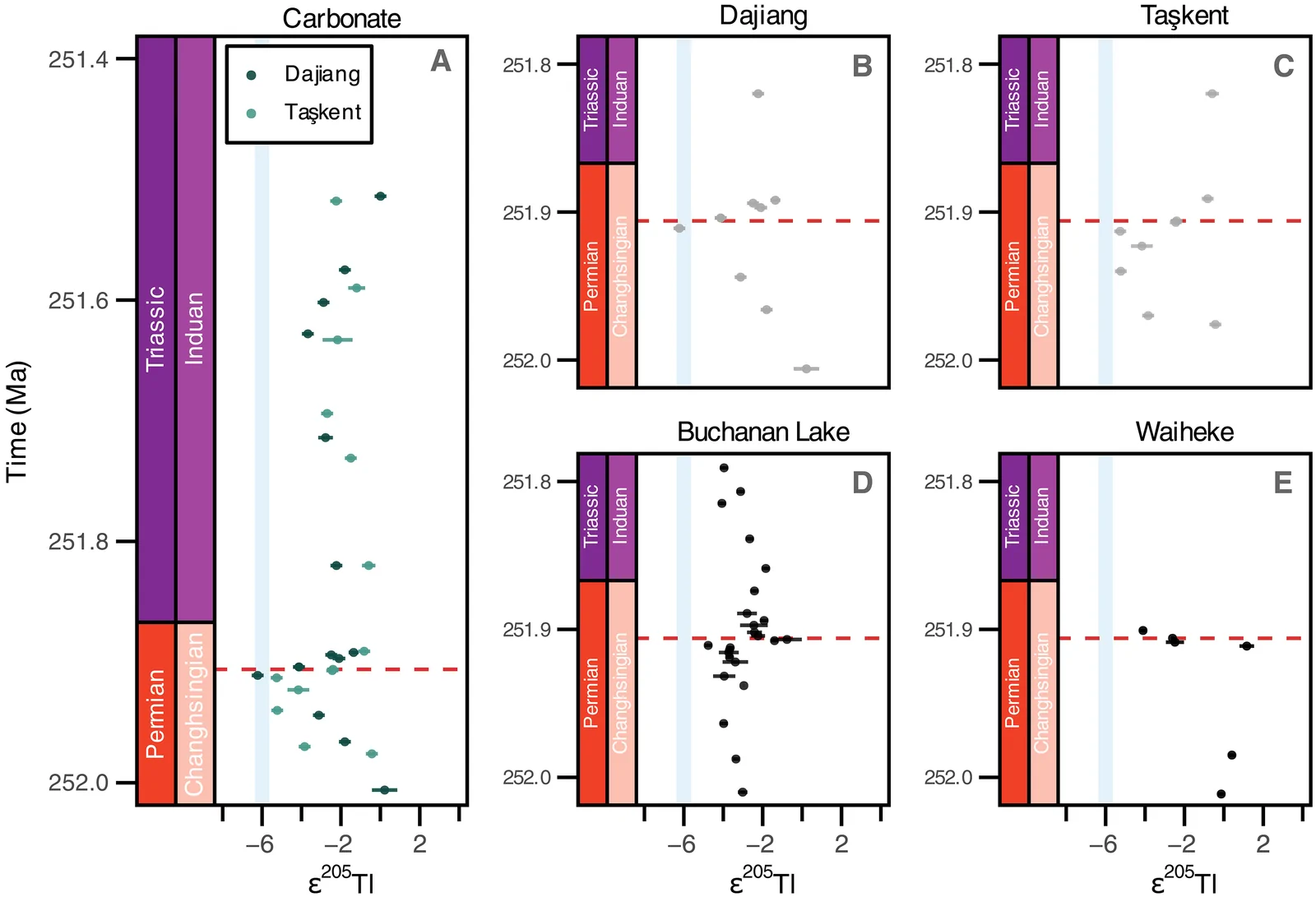
Time-correlated carbonate ε^205^Tl record and magnified views of the PTB for both the shale and carbonate sections. (**A**) Carbonate ε^205^Tl records from the Dajiang and Taşkent sections on the same timescale and zoomed-in plots of the four new records generated here, including the carbonate (**B**) Dajiang and (**C**) Taşkent sections and the shale-dominated (**D**) Buchanan Lake and (**E**) Waiheke sections, more clearly demonstrating the brief pre-extinction negative excursion and following shift toward more positive ε^205^Tl values across the EPME horizon. The light blue lines represent the ε^205^Tl of modern, well-oxygenated seawater, and the red dashed lines mark the EPME [∼251.906 Ma (*1*)]. Error bars represent ±2 SD; where not visible, they are smaller than the plotted symbols.

Despite the general similarity in trends between sections, especially during key intervals such as directly before and after the EPME (Fig. 5, B to E), there is also variability in the overall record. All four sections include several ε^205^Tl values higher than −2‱, the average crustal input value. These values are difficult to reconcile with the conventional mass-balance framework, which suggests that an ocean without Mn oxide burial would result in ε^205^Tl values of −2‱ (Fig. 1C) and offers no explanation for values exceeding this threshold. These anomalously high values could result from diagenetic alteration of the ε^205^Tl signal or local depositional factors, such as the inclusion of Mn oxides. Trace-element analyses of the carbonate samples revealed no obvious correlations between Mg/Ca, Mn/Sr, Mn, Tl, or Al and Th concentrations with ε^205^Tl or Mn concentrations with Tl (fig. S1). However, examining these correlations cannot rule out contributions of Tl from Mn oxides that were subsequently removed by anoxic diagenesis, releasing Tl that could be partially recaptured by trace sulfides and decoupled from Mn concentrations. Weathering could also potentially shift ε^205^Tl values higher, especially in samples collected from outcrop (*69*), although we do not have reason to believe that any of our samples were substantially weathered on the basis of observations made in the field and δ^18^O values of the carbonate samples (*15*, *63*) (see also data S1). At Waiheke, nearly all of the pre-extinction ε^205^Tl measurements fall above −2‱ (Figs. 2, 3, and 5E). This finding may indicate mildly oxidizing local redox conditions at this site for part of the Late Permian (*19*), which could have facilitated a minor contribution from ^205^Tl-rich Mn oxides [cf. modern upwelling zones; (*49*)]. Much of the variability among sections may also result from poor age constraints in some of the measured sections. Alternatively, widespread anoxia may have drawn down the seawater Tl reservoir sufficiently to reduce Tl’s residence time below the global ocean mixing time (∼1 kyr) (*30*), resulting in only partial homogenization of Tl isotope ratios among basins.

### Evidence for pre-extinction deoxygenation

Previous interpretations of local redox conditions for Permian-Triassic sites have suggested that anoxic conditions may have initiated in the deep sea (*17*, *19*, *70*, *71*). Evidence for globally extensive deep-ocean anoxia preceding the EPME was reported in the first Permian-Triassic Tl isotope study (*18*). Our age calibration of eight Permian-Triassic ε^205^Tl records allows us to better constrain the timing and duration of widespread deoxygenation.

According to our age calibrations (Fig. 3), ε^205^Tl values from all sections are higher than the modern, well-oxygenated ε^205^Tl value of −6‱ from ∼253.0 to 252.1 Ma, suggesting that Late Permian oceans were already broadly oxygen depleted during this interval (Fig. 4). Wang *et al.* (*60*) also reported Tl isotope evidence for deoxygenation preceding the main extinction pulse based on their results from the Meishan section, although they suggested an onset only 30 kyr before the EPME. This discrepancy likely reflects the limited temporal coverage of the Late Permian in their dataset rather than a fundamental disagreement between records. Although not displayed here due to a lack of age constraints, two Capitanian-aged Tl isotope measurements from the Opal Creek section suggest that the oceans had been well oxygenated (ε^205^Tl < −6‱) earlier in the Late Permian (*18*).

This ε^205^Tl evidence of deoxygenation challenges U isotope evidence suggesting a well-oxygenated latest Permian ocean with the onset of anoxia coinciding with or slightly preceding the extinction (*14*–*16*, *21*, *23*, *72*, *73*). Uranium removal and isotope fractionation are the most pronounced today in shelf and slope environments and euxinic basins, where the combined effects of organic carbon burial and sulfide accumulation promote U(VI) reduction (*74*). Sediments from deep-ocean settings, by contrast, do not efficiently remove U and drive comparatively small isotope fractionation effects, likely due to low productivity (*25*). Although U-based reconstructions provide valuable constraints on oxygen availability in high-productivity shelf and slope environments, they may be less sensitive to episodes of seafloor anoxia in the deep ocean. Furthermore, the higher redox potential (Eh) of Mn(II) oxidation (and therefore the Tl isotope proxy) compared to U(VI) reduction may indicate that Tl isotopes begin to respond under more weakly reducing conditions than those required to affect U isotopes. It is possible that, for the Late Permian, Tl isotopes record an increase in global ocean deoxygenation that was either too subtle to be detected by U isotopes, confined to the deep ocean where deoxygenation is thought to have initiated (*70*) or a combination of these scenarios.

### Reoxygenation before the EPME

Another notable feature of the records presented here and of those from Newby *et al.* (*18*) and Wang *et al.* (*60*) is the presence of a negative excursion to modern or near-modern ε^205^Tl values immediately before the extinction. Minimum pre-extinction ε^205^Tl values are −6.2‱ at Dajiang, −5.3‱ at Taşkent, and −4.8‱ at Buchanan Lake. At Waiheke, we found a pre-extinction excursion of similar magnitude, although due to higher starting values, it reaches a minimum of only −2.6‱ before the EPME. However, just 2 cm above the likely extinction horizon, in the same black shale bed, ε^205^Tl decreases to −4.1‱. This excursion was also reported by Newby *et al.* (*18*), who documented a negative shift in ε^205^Tl to values below −6‱ immediately preceding the extinction in the Ubara and Opal Creek sections, and by Wang *et al.* (*60*), who also reported ε^205^Tl values near −6‱ directly before the EPME in the Meishan section. The presence of this negative excursion in seven globally distributed and age-calibrated sections increases confidence that this is a robust global signal caused by a brief reoxygenation on the timescale of tens of thousands of years preceding the extinction event.

Newby *et al.* (*18*) hypothesized that a transient ocean oxygenation was triggered by widespread sulfate aerosol release from the Siberian Traps, causing climatic cooling and thus enhancing ocean circulation and O_2_ solubility. Increased O_2_ in seawater would promote Mn oxide burial, preferentially removing ^205^Tl and leaving seawater enriched in ^203^Tl, resulting in a negative isotope excursion. Although we also interpret the notable changes in Permian-Triassic Tl isotopes to be predominantly driven by trends in oxygenation, the Tl cycle can also be perturbed (and more directly) on millennial timescales by changes to the Mn cycle (*29*, *75*) that can occur rapidly due to the short residence time of Mn in the ocean (*76*). Although such perturbations of the Mn cycle may well be related to changes in ocean oxygenation, these perturbations result in non-steady state conditions for the marine Tl isotope budget (*29*). If large parts of the ocean become strongly depleted in oxygen, as may have been the case in the period leading up to the EPME, the additional dissolved Mn that builds up provides potential for a subsequent short period of enhanced Mn oxide precipitation that could be triggered by relatively modest ocean oxygenation without requiring a fundamental rearrangement of the marine redox structure. The temporal resolution of our data does not allow us to determine whether some of the more negative values leading up to the EPME were caused by millennial-scale perturbations of the Mn cycle. However, the potential for such processes to have occurred imply that, although some level of reoxygenation most likely occurred, it may not have been to modern levels because of the possible decoupling between the marine Mn cycle and oxygen contents (*75*). The model fit of the Tl isotope data does not reconstruct a full return to the modern seawater ε^205^Tl values of −6‱ (Fig. 4A). Instead, it is consistent with a limited shift in ε^205^Tl_sw_ caused by a disproportionately large flux of Mn oxide burial, as evidenced by the nonlinear relationship between ε^205^Tl and the fraction of the seafloor conducive to Mn oxide burial (Fig. 1C).

Following this apparent reoxygenation, all of our new records and most of those from Newby *et al.* (*18*) and Wang *et al.* (*60*) exhibit a sharp positive excursion across the PTB (or directly before it). Geochemical modeling suggests this excursion reflects a decrease in the amount of Mn oxide burial for at least the next 600 kyr (and possibly much longer), most likely caused by a return of widespread anoxia (Fig. 4). Some of our sensitivity tests, such as those using ε^205^Tl data from all available Permian-Triassic records or from just the siliciclastic-dominated sections, indicate that the return to widespread anoxia occurs in the Early Triassic, around ∼251.7 Ma (fig. S3), although it is important to note that both of these tests include the potentially diagenetically altered record from Waiheke.

Both Tl and U isotopes record a shift toward deoxygenation during this interval. This finding is consistent with the widespread onset of euxinia on shelf and slope environments seen in the Early Triassic (*11*, *15*, *22*, *77*) and suggests that postextinction anoxia was more extensive and/or more pronounced in shallow-marine environments. When the ocean is already well-ventilated and the Mn reservoir is small, Tl isotopes are an excellent tracer of changes in deep-ocean deoxygenation. However, when large areas of the deep ocean are anoxic, such as we expect was the case in the latest Permian, the Mn content of the ocean can rise. In this scenario, Tl isotopes may become more sensitive to changes in oxygenation in shallow-marine environments. This prolonged instability of seafloor oxygenation is consistent with other evidence for protracted anoxia [e.g., (*15*, *23*)] and may provide insight into one potential cause of the delayed biotic recovery observed in the fossil record (*78*, *79*).

### Paired carbonate and shale ε^205^Tl records

The similarities between the carbonate and shale records developed here strengthen the case for the recently proposed applicability of the ε^205^Tl paleoredox proxy to the ancient carbonate rock record. The visual similarities between the carbonate and shale records were further tested using sensitivity analyses to assess whether model runs of the separated carbonate and shale records produce similar predictions of *f*_mnox_. We found good agreement between the records when modeled independently, both predicting widely dysoxic Late Permian oceans for the ∼1 Myr before the extinction, interrupted by a brief reoxygenation of similar magnitude (*f*_mnox_ reaching 99% in shales and 98% in carbonates) directly before the EPME, followed by a return to widely deoxygenated conditions (fig. S4).

Our results show the Tl isotope proxy holds considerable promise for ancient carbonate rocks. Until the approach is more fully validated, best practice requires analyzing multiple carbonate sections, preferably from different basins, to ensure robust results. Ideally, carbonate ε^205^Tl records would be paired with those from low-oxygen siliciclastic sequences, which are currently better established to capture seawater Tl isotope values across many intervals of geologic time (*29*, *32*–*39*). The processes that diagenetically alter carbonates and shales differ due to their different depositional environments and likely host minerals for Tl, reducing the likelihood that they will be correlated due to secondary alteration rather than changes in the primary ε^205^Tl seawater signal. Carbonates are highly reactive with fluids and are prone to dissolution, recrystallization, and authigenic (porewater) carbonate precipitation (*80*), whereas diagenesis in shales typically results from compaction and postdepositional redox reactions (*81*). To a first order, consistent carbonate-shale Tl isotope records can increase the evidence that the values are globally representative of the seawater ε^205^Tl value.

The Tl isotope proxy is uniquely powerful because of its applicability to both carbonate and siliciclastic lithologies without requiring assumptions about differing magnitudes of fractionation relative to seawater. Other global redox proxies (e.g., δ^238^U and δ^98^Mo) cannot be used in both carbonates and shales to directly measure seawater isotope signals. Although the U isotope proxy was originally developed for use in shales (*82*), its application is complicated by the need to correct for detrital U (*83*) and the large variability in isotope fractionation effects observed across the type of locally reducing sites today that later become organic-rich mudrocks (*74*, *84*). Conversely, δ^98^Mo presents challenges when used in carbonate rocks because biogenic calcite can be variably offset from seawater (*85*). Furthermore, δ^98^Mo measured in modern carbonate settings rarely reflects the seawater composition (*86*). Paired ε^205^Tl records from carbonates and shales increase our ability to compare sections from geographically distant and environmentally distinct regions. This dual applicability enables the development of continuous records in sections that alternate between siliciclastic and carbonate lithologies.

### Implications for the EPME

Deoxygenation occurred both before and after the EPME (*11*–*21*, *72*), although its relative importance as a kill mechanism has been difficult to assess given uncertainty regarding the spatial extent and distribution of marine anoxia. Uranium isotope records from the PTB are interpreted to represent the expansion of oxygen minimum zones (OMZs) onto shelves in the Early Triassic (*15*), but this proxy may not capture global deoxygenation in deeper environments. To develop a complete picture of seafloor anoxia during the EPME, it is also necessary to use proxies that provide insights into deep-ocean redox conditions, which Tl isotopes achieve by tracking the shutoff or slowing of Mn oxide burial. Our modeling results suggest that deep environments may also have experienced deoxygenation in both the Late Permian and Early Triassic, consistent with abyssal plains sedimentary records (*17*, *19*, *70*, *71*, *87*). This Late Permian, pre-extinction deoxygenation was likely noneuxinic and limited to the deep ocean, which could be a result of changes in global ocean circulation patterns that may have slowed down delivery of dissolved O_2_ to the deep ocean from high latitudes (*29*). Deep environments may have experienced some extinction during this million-year interval (*70*, *88*), yet this did not approach the severity of the EPME, in part due to the already limited biodiversity of the deep sea and perhaps also due to the relatively cooler and stable deep-water temperatures. In contrast, the deoxygenation that developed during and after the EPME was likely strongly euxinic and affected highly populated shallow-marine environments (*77*), which, when coupled with the extreme increases in temperature in the Early Triassic (*8*), led to much of the marine diversity loss we observe in the fossil record (*10*, *65*, *68*). Reconstructing the full extent of these conditions requires integrating Tl isotope records with other paleoredox proxies of varying spatial sensitivities. Together, they help bring the circumstances of Earth’s greatest mass extinction event into focus.

## MATERIALS AND METHODS

### Experimental design

#### Geologic setting and age models

We analyzed two carbonate and two shale-dominated sections from four geographically distinct regions to assess whether the ε^205^Tl records are reproducible globally and across lithologies (Fig. 1A). The carbonates analyzed come from the Dajiang section on the Great Bank of Guizhou, South China, from the eastern Tethys, and the Taşkent section from within the Aladag Nappe, Türkiye, from the western Tethys. Both sections document tropical, shallow-marine carbonate platforms from equatorial latitudes. We also analyzed mudstone samples from the Buchanan Lake section on Axel Heiberg Island in the Sverdrup Basin of Canada and the Waiheke section at Island Bay on Waiheke Island, North Island, New Zealand, representing northern and southern Panthalassa, respectively. These two mudstone sections document deeper-water conditions at mid-latitudes.

Samples from the Dajiang, Taşkent, and Buchanan Lake sections were assigned estimated ages based on conodont and foraminiferan biostratigraphy and δ^13^C chemostratigraphy using the Late Permian age model of Burgess *et al.* (*89*) and the Early Triassic age model of Dai *et al.* (*1*). Samples from Waiheke were assigned ages based on a calculated sedimentation rate of 3.8 m/Myr (*19*). Ages were also assigned to the Opal Creek, Gujo-Hachiman, and Ubara ε^205^Tl records from Newby *et al.* (*18*) using calculated sedimentation rates and bio-, chemo-, and magnetostratigraphy (*90*, *91*) and to the Meishan record from Wang *et al.* (*60*) using radiometric U-Pb dates from volcanic ash beds as well as bio- and chemostratigraphy (*1*, *60*, *89*, *92*, *93*). A more detailed description of the geologic setting, including construction of the age models and prior geochemical studies from these locations, can be found in the Supplementary Materials, and specific tie points used are provided in data S1.

#### Trace-element analysis

Carbonate trace-element data were generated in the inductively coupled plasma mass spectrometry (ICPMS) lab at the University of Utah. A small amount of powdered sample, about 0.05 g, was placed in a trace-metal-clean centrifuge tube and slowly amended with 10 ml of 1 M nitric acid, gently capped, and left overnight at room temperature. Samples were thereafter centrifuged, and the supernatant transferred to a new clean tube. An Agilent 8900 was used to quantify trace-metal concentrations for a small supernatant aliquot. Concentrations were quantified using an external calibration method with indium added as an internal standard. Certified Reference Material 1643f (Trace Elements in Water, National Institute of Standards and Technology, USA) was used repeatedly to assess the quality of the calibration curve prepared from mixing single-element standards (Inorganic Ventures, USA). Measured concentrations were reproduced within 5% of the certified values.

#### Thallium isotope analysis

Carbonate samples were bulk digested because bulk digests of modern carbonates yield ε^205^Tl values analytically indistinguishable from seawater, whereas selective leaches do not (*1*). Samples were first dissolved in 8 M nitric acid, and any remaining residues were fully digested by heating in a series of concentrated nitric, hydrochloric, and hydrofluoric acid mixtures. Shale samples were partially digested using the methods of Nielsen *et al.* (*40*) and Owens *et al.* (*41*). The digested samples were put through a two-step ion-exchange chromatography following the methods of Nielsen *et al.* (*94*). Carbonate Tl isotope ratio measurements were performed on a Neptune Plus located at the University of Utah ICPMS facility, and shale Tl isotope ratios were measured on a Thermo Fisher Scientific Finnigan Neptune multicollector ICPMS (MC-ICPMS) located at the Woods Hole Oceanographic Institution (WHOI) Plasma Facility. In-depth analytical methods can be found in the Supplementary Text.

### Statistical analysis

Trace-element data were used to test diagenetic alteration of the Tl isotope ratio in the carbonate samples. Many different elements (Tl and Mn) and element ratios (Mg/Ca and Mn/Sr) were plotted against each other and against ε^205^Tl to check for potential correlations (see Results for additional information). We used Spearman’s ρ to determine the strength of correlations

$$\rho =1-\frac{6{\Sigma d}^{2}}{n\left(n^{2}-1\right)}$$ (2)

where *d* is the difference between the ranks of *X* and *Y* and *n* is the total number of observations. We define weak correlations as ρ ≤ 0.39, moderate correlations as 0.40 ≤ ρ ≤ 0.59, and strong correlations as ρ ≥ 0.60. We evaluated statistical significance by calculating *P* values using the cor.test function in R and consider *P* ≤ 0.05 as statistically significant.

## Supplementary Materials

### The PDF file includes:

Supplementary Text Figs. S1 to S4 Table S1 Legend for data S1 References
