# Supplementary material for: Coupled carbonate-shale thallium isotopes reveal the evolution of deep-marine anoxia during the end-Permian mass extinction
Source: Sci Adv. 2026 Oct 28;12(44):eaef5669. doi: 10.1126/sciadv.aef5669 (PMC13715695; doi:10.1126/sciadv.aef5669)
Supplement: Supplementary file 1 [file sciadv.aef5669_sm.pdf]

Science Advances
AAAS

Supplementary Materials for

Coupled carbonate-shale thallium isotopes reveal the evolution of deep-marine anoxia during the end-Permian mass extinction

Susannah C. Herz *et al.*

Corresponding author: Susannah C. Herz, herz@stanford.edu; Menghan Li, mhli@must.edu.mo

Sci. Adv. **12**, eaef5669 (2026)
DOI: 10.1126/sciadv.aef5669

The PDF file includes:

Supplementary Text
Figs. S1 to S4
Table S1
Legend for data S1
References

Other Supplementary Material for this manuscript includes the following:

Data S1

Supplementary Text

Geologic Setting

Four globally distributed geologic sections were examined here for ε^{205}Tl. The two carbonate sections analyzed come from geographically separated, tropical, shallow-marine platform environments. The Dajiang section (25°33′59″ N, 106°39′36″ E) is located on the Great Bank of Guizhou (GBG), an isolated carbonate platform in the Nanpanjiang Basin of the equatorial eastern Tethys. The Dajiang section preserves 2500 m of Permian-Triassic, shallow-marine strata from the platform interior (65). The GBG has a detailed geochronology and chronostratigraphy developed from conodont and foraminifera biostratigraphy, paleomagnetic reversal data, and carbon isotope chemostratigraphy (96–98). Many geochemical studies have been conducted on the Dajiang section, including investigations of carbon, calcium, uranium, molybdenum, and strontium isotopes (15, 20, 63, 95, 99–104), in addition to numerous stratigraphic and paleontological studies (65, 98, 105–108). The Dajiang ε^{205}Tl record includes 14 samples spanning 97.9 m of stratigraphy from the Changhsingian through Induan stages.

The Taşkent section (36°51′ N, 32°30′ E) is within the Aladag Nappe, Türkiye, and preserves ~1000 m of carbonate and siliciclastic shallow-marine sediments from the low-latitude western Tethys, spanning the uppermost Permian to Middle Triassic (95). Carbon, calcium, oxygen, and uranium isotope records have been generated for the Taşkent section (15, 95). This area is temporally well-constrained through chemostratigraphic correlations of δ^{13}C to the GBG and first appearances of foraminifera and conodonts (15). The ε^{205}Tl record for Taşkent comprises 14 samples from 165.8 m of Changhsingian through Induan strata.

Black shales spanning the Upper Permian to Lower Triassic were sampled from the Buchanan Lake section (79°26′6″ N, 87°12′36″ W) on Axel Heiberg Island in the Sverdrup Basin of Canada. This section documents deep-water conditions in a mid-latitude region of the Panthalassa Ocean (12). The Buchanan Lake section has been the subject of detailed geochemical studies, including stable carbon isotopes of organic matter, organic petrography, and nitrogen, molybdenum, mercury, and nickel stable isotope records (12, 22, 58, 62, 109–111). Redox-sensitive trace metal enrichments revealed local anoxia in the ~38 m of strata preceding the EPME (12). Further studies found δ^{15}N > 9 ‰ and high concentrations of lycopane biomarkers, which suggest that local conditions were at least suboxic throughout the deposition of 85 m of strata before the extinction (58). Estimated ages were assigned to the Buchanan Lake samples using δ^{13}C chemostratigraphy and the age model of Dai *et al*. (1). The 50 Buchanan Lake samples in the ε^{205}Tl record span 66.3 m and ~950 kyr of time.

Additional mudstone samples were collected from the Waiheke section (36°46′8″ S, 175°0′12″ E) at Island Bay on Waiheke Island, North Island, New Zealand. In the Permian-Triassic interval, the Waiheke section was in southern Panthalassa at a paleolatitude of ~34°S, as predicted by paleomagnetic data (112). This section documents deep-water deposits of siliceous mudstones and black shales (17, 19, 67). At Waiheke, increasingly ferruginous anoxic conditions through the Late Permian were detected using iron speciation and redox-sensitive trace metal enrichments (19). Age estimates for the Waiheke section are based on a detailed conodont biostratigraphy (17), δ^{13}C chemostratigraphy (17, 67), and a calculated sedimentation rate of 3.8 m/Myr (19). Existing geochemical records for Waiheke include organic carbon, mercury, and sulfur isotopes, as well as trace metal, pyrite framboid, and iron speciation data (17, 19, 67, 113). Thallium isotope data were collected for 11 samples from Waiheke, all of which are Changhsingian in age due to low Tl concentration in most samples above the extinction horizon.

The Opal Creek, Gujo-Hachiman, and Ubara sections from the work of Newby *et al*. (18) were also assigned ages for their inclusion in the data compilation used in modeling. The Gujo-Hachiman and Ubara sections were the subject of detailed stratigraphic analyses from Algeo *et al*. (90). Using Milankovitch cyclicity, radiolaria and conodont biostratigraphy, magnetostratigraphy, and chemostratigraphy, key events such as the end-Permian Mass Extinction and Permian-Triassic boundary were defined, allowing estimated sedimentation rates to be calculated throughout the sections (90). The Opal Creek section also has a well-developed conodont biostratigraphy (91). Using the age estimates of Dai *et al*. (1) for the end-Permian Mass Extinction (Bed 25 in the Meishan section, South China) and the first appearance datum (FAD) for *Hindeodus parvus*, we interpolated ages for the rest of the section assuming a linear sedimentation rate. Similarly, ages were assigned to samples from the Meishan section following Wang *et al*. (60). Meishan is the Global Boundary Stratotype Section and Point for the Permian-Triassic boundary, and as such has exceptional age constraints, including U-Pb dates from ash beds and absolute ages assigned to important bio- and chemostratigraphic markers (1), which were utilized here. The tie points used to create the age models for each of the eight sections are available in the *Supplemental Data*.

Thallium Isotope Analysis

Preparation of shale samples for Tl isotope analyses took place at Woods Hole Oceanographic Institution (WHOI) in the NIRVANA class 100 trace metal clean lab. For carbonate samples, preparation took place at the University of Utah in a generic geochemistry lab using HEPA filter laminar flow boxes and exhausted hoods. Acids used in both locations were trace metal grade (at least), and all sample vessels were pre-cleaned with these acids prior to use.

Routine Tl isotope ratio analyses require at least a few nanograms of Tl (ideally, at least 15 ng; 94, 114). For relatively Tl-rich shales, this amount of authigenic Tl can be recovered from ~0.1 g of powdered material. Approximately 0.1 g of powdered shale material was weighed into perfluoroalkoxy alkane (PFA) vials and amended with 3 mL of 2 M nitric acid on a hot plate for 12-15 hours at 130 °C. This method has been shown to effectively isolate primarily sulfide-bound authigenic Tl from terrigenous Tl (40, 41). After heating, the authigenic and terrigenous fractions were separated via centrifugation. Supernatant authigenic fractions were transferred into PFA vials for complete digestion; the terrigenous fractions were set aside. Complete authigenic digestion was achieved with a series of concentrated nitric and hydrochloric acid mixtures on a hot plate. Samples with high organic contents required repeated acid attacks with concentrated nitric acid and hydrogen peroxide.

Recovering requisite Tl from the Tl-lean carbonate samples required ~2 g of powdered material. Carbonate samples were crushed to fine powders with a porcelain mortar and pestle. Care was taken to remove weathered surfaces and veins prior to crushing. Approximately 2 g of powdered carbonate material was weighed into 50 mL centrifuge tubes, slowly amended with 25 mL of ~8 M nitric acid, and left gently capped overnight to fully dissolve carbonate minerals. The supernatants were then transferred to new 50 mL centrifuge tubes and set aside; solid residues were transferred to PFA vials and digested independently. Complete digestion of the solid residues was achieved with a series of concentrated nitric, hydrochloric, and hydrofluoric acid mixtures on a hot plate. Once completely digested, the solid residue fractions were recombined with the authigenic fractions, based on the finding from Shu *et al*. (44) that bulk carbonate digests best match modern seawater Tl isotope values. This methodology varies from

that used by Shu *et al.* (44) due to differences in the samples. Compared to the modern and recent carbonates from the Bahamas, the Permian-Triassic carbonates were more well-lithified and less $CaCO_3$ rich, making them more resistant to digestion.

After digestion, shale and carbonate samples were amended with 1 M HCl and brominated water in preparation for two-step ion exchange chromatography (47). For the shales processed at WHOI, samples were passed through large glass columns and then small Teflon columns. For the carbonates processed at the University of Utah, samples were passed twice through plastic columns (Bio-Rad), first with 0.7 mL and then 0.3 mL of AG1X8 anion exchange resin. After each column step, samples were treated with concentrated nitric acid and hydrogen peroxide and heated on a hot plate at 130°C for at least 4 hours to digest organics originating from the resin that was eluted with the Tl. After chromatography, samples were constituted in 0.5 mL of 0.1 M nitric acid plus 0.1% sulfuric acid in preparation for isotope ratio measurements.

Shale Tl isotope ratio measurements were performed on a Thermo Finnigan Neptune multi-collector ICPMS (MC-ICPMS) equipped with an Aridus II desolvating nebulizer sample introduction system located at the WHOI Plasma Facility. Carbonate Tl isotope ratio measurements were performed on a Neptune Plus equipped with an Aridus III located at the University of Utah ICPMS facility. The same analytical methods were used for both machines (32, 46). Measurements were performed in low-resolution mode using standard-sample bracketing and external normalization to NIST SRM 981 Pb. Because the ion exchange separation procedure returns 100% yields (32, 46) and each sample was doped with a known quantity of NIST SRM 981 Pb, Tl concentrations could be calculated during MC-ICPMS analysis using measured $^{205}Tl/^{208}Pb$ ratios. A Sigma Aldrich Tl standard solution measured on the University of Utah instrument (–0.7 ± 0.4‱; 2SD; n = 10) yielded $\varepsilon^{205}Tl$ values within analytical uncertainty of those measured for the same solution on the WHOI instrument (–0.8 ± 0.4‱; 2SD; n = 2). These Aldrich $\varepsilon^{205}Tl$ values are consistent with those reported in previous work (2). USGS shale SCo-1 was processed with each batch of shale samples to monitor accuracy and yielded $\varepsilon^{205}Tl$ values (–3.0 ± 0.3‱ 2SD; n = 3) within analytical uncertainty of previous work (–2.9 ± 0.1‱; 2SD; 54). The NCS DC 70307 carbonate rock standard was processed with each batch of carbonate samples and yielded an average bulk-rock $\varepsilon^{205}Tl$ value of 0.2 ± 0.8‱; 2SD (n = 4).

Thallium Isotope Inverse Model

A Bayesian inverse model of Tl isotopes was used to infer quantitative changes in the extent of seafloor conducive to manganese oxide burial throughout the study interval. The Tl model was adapted from the inverse model of the U cycle from Kipp and Tissot (24), where the fraction of seafloor anoxia (f_{anox}; modern = 0.00211) is used to drive observed changes in $\delta^{238}U$. An inverse model enables objective, quantitative assessment of model-data fit through statistical optimization, eliminating the subjective parameter tuning inherent in forward model approaches that solely rely on ad hoc multiple forward model runs. Importantly, this model is dynamic, allowing the ocean mass balance to evolve through time, rather than assuming steady-state conditions throughout (*24*).

Here, we follow a similar approach to relate reconstructed $\varepsilon^{205}Tl_{sw}$ values to the fraction of seafloor that is oxygenated enough to support Mn oxide burial (f_{mnox}). The Tl output fluxes (i.e. low-temperature basalt alteration, reducing sediments, and Mn oxide burial) were defined according to the framework outlined by Lau *et al*. (15) for the U system:

$$J_{basalt} = K_{basalt} \times N_{sw} \quad (2)$$
$$J_{anox} = K_{anox} \times N_{sw} \times f_{anox} \quad (3)$$
$$J_{mn\text{-}oxide} = K_{mn\text{-}oxide} \times N_{sw} \times (1 - f_{anox}) \quad (4)$$

where K_{basalt}, K_{anox}, and $K_{mn\text{-}oxide}$ are rate constants for each of the outputs, N_{sw} is the total amount of Tl in seawater (in mols), and f_{anox} is the fraction of the seafloor that is lacking sufficient O_2 to sustain Mn oxide stability in sediments; consequently, f_{mnox} is the complementary fraction that remains oxygenated (Equation 5). Using the input flux (J_{in}, in mols Tl yr^{-}), the rate constants were calculated according to the mass balance equation to ensure the outputs began at the correct initial proportions (65%, 5%, and 30%, respectively; 115) (Equations 6, 7, 8):

$$f_{mnox} = 1 - f_{anox} \quad (5)$$
$$K_{basalt} = 0.65 \times J_{in}/N_{sw} \quad (6)$$
$$K_{anox} = (J_{in} - K_{basalt} \times N_{sw})/(N_{sw} \times f_{anox} + 0.0125 \times N_{sw} \times (1 - f_{anox})) \quad (7)$$
$$K_{mn\text{-}oxide} = 0.0125 \times K_{anox} \quad (8)$$

The results of these calculations can be found in Table S1, along with the rest of the parameter values used in the initial steady state. These relationships were also used to establish an ordinary differential equation (ODE) (Equation 9) that could be run in the forward model. A 200-kyr spin-up period allowed the model to be initialized with the modern mass balance for each system and progress toward the initial states. The evolution of f_{anox} through time is represented as a Fourier series, a sum of sine and cosine terms whose coefficients set the amplitude and period of their oscillations (see 24). Markov Chain Monte Carlo (MCMC) simulations were used to iteratively run the forward model, sampling these Fourier series coefficients to simulate the evolution of seawater oxygenation through time. Using the Metropolis-Hastings algorithm (116, 117), parameter sets that better match the observed data are preferentially retained. Here, we use a data compilation from the five ε^{205}Tl records that extend into both the pre- and post-extinction intervals and are likely to be unaltered (Dajiang, Taşkent, Buchanan Lake, Gujo-Hachiman, and Meishan). We also ran tests using data from all eight records, removing data points > −2‱, and using an input value of 0‱ (see *SI Modeling Sensitivity Analysis*).

$$d\varepsilon^{205}Tl_{sw}/dt = (J_{in}*(\varepsilon^{205}Tl_{in} - \varepsilon^{205}Tl_{sw}) - J_{anox}*\Delta_{anox} - J*\Delta_{mn\text{-}oxide} - J_{basalt}*\Delta_{basalt})/N_{sw} \quad (9)$$

The model's cost function (i.e., the criterion used to evaluate the fit between predicted and observed values) requires each measurement to have an associated uncertainty. In addition to analytical error, each sample carries uncertainty relative to the seawater value from local rock-related variation, assumed to reflect random (Gaussian) deviation from the seawater signal. Here, this variability exceeds the analytical error despite having samples spaced substantially more closely than the residence time of Tl in seawater. We calculated the standard deviation of the differences between each stratigraphically consecutive ε^{205}Tl value (following Pimentel-Galvan *et al*. (23)) and used this as a representative measure of uncertainty for each sample relative to the true value of seawater. We additionally allow the model to propagate uncertainty on inputs typically held constant (e.g., the rate constants and fractionations associated with each of the inputs and outputs of ε^{205}Tl).

Model Sensitivity Analysis

We performed a series of sensitivity analyses on the Tl isotope inverse model to evaluate the model's ability to fit many data points that are higher than the average crustal input value of −2‱. According to the conventional Tl isotope mass balance, values of −2‱ indicate that 0% of the seafloor can support Mn oxide burial. Under these conventional mass balance assumptions, the model contains no mechanism that could produce heavier values. To test the impact of our unusually heavy ε^{205}Tl values on modeling interpretations, we compared results from model runs 1) using the conventional Tl mass balance (inputs = −2‱), 2) removing all ε^{205}Tl data points higher than −2‱, and 3) using an adjusted (isotopically heavier) input value (inputs = 0‱) (Figure S3).

Using the conventional mass balance, we also compared the results of using only the five sections that have substantial data both before and after the extinction and are likely unaltered (Dajiang, Taşkent, Buchanan Lake, Gujo-Hachiman, and Meishan) and using all eight sections. We found that the model still reconstructed a substantial increase in f_{mnox} immediately prior to the extinction when using data from all sections, although it shifts the return of widespread deoxygenation to the Early Triassic rather than across the extinction. When removing all data points higher than −2‱, the model indicates that the extent of well-oxygenated seafloor is variable leading up to the extinction, but still somewhat depleted from ~252.9 Ma until the brief reoxygenation event preceding the EPME. We performed a model test using an ε^{205}Tl input value of 0‱ to see if this could help account for some of the higher data points. This change did not prevent the model reconstruction from predicting that 0% of the seafloor can support Mn oxide burial for part of the Late Permian. However, this change to the mass balance did shift the ε^{205}Tl value corresponding to f_{mnox}= 1 to −4‱ (rather than the modern −6‱), resulting in model predictions of far less deoxygenation through the end of the Late Permian and Early Triassic.

We also compared results from model runs fitting the carbonate and shale ε^{205}Tl records separately to see if data from different lithologies produces similar estimates of deoxygenation (Figure S4). Both the carbonate-only and shale-only model runs predict f_{mnox} = 0 during the Late Permian. They both also reconstruct a brief reoxygenation immediately prior to the extinction, to 0.99 and 0.98 f_{mnox} for shales and carbonates, respectively. This reoxygenation is followed by a return to f_{mnox} = 0 in the Early Triassic for shales and coinciding with the extinction for carbonates. The similarity in results produced by each separate lithology provides confidence that the ε^{205}Tl proxy is working to record primary seawater signals in carbonate rocks.

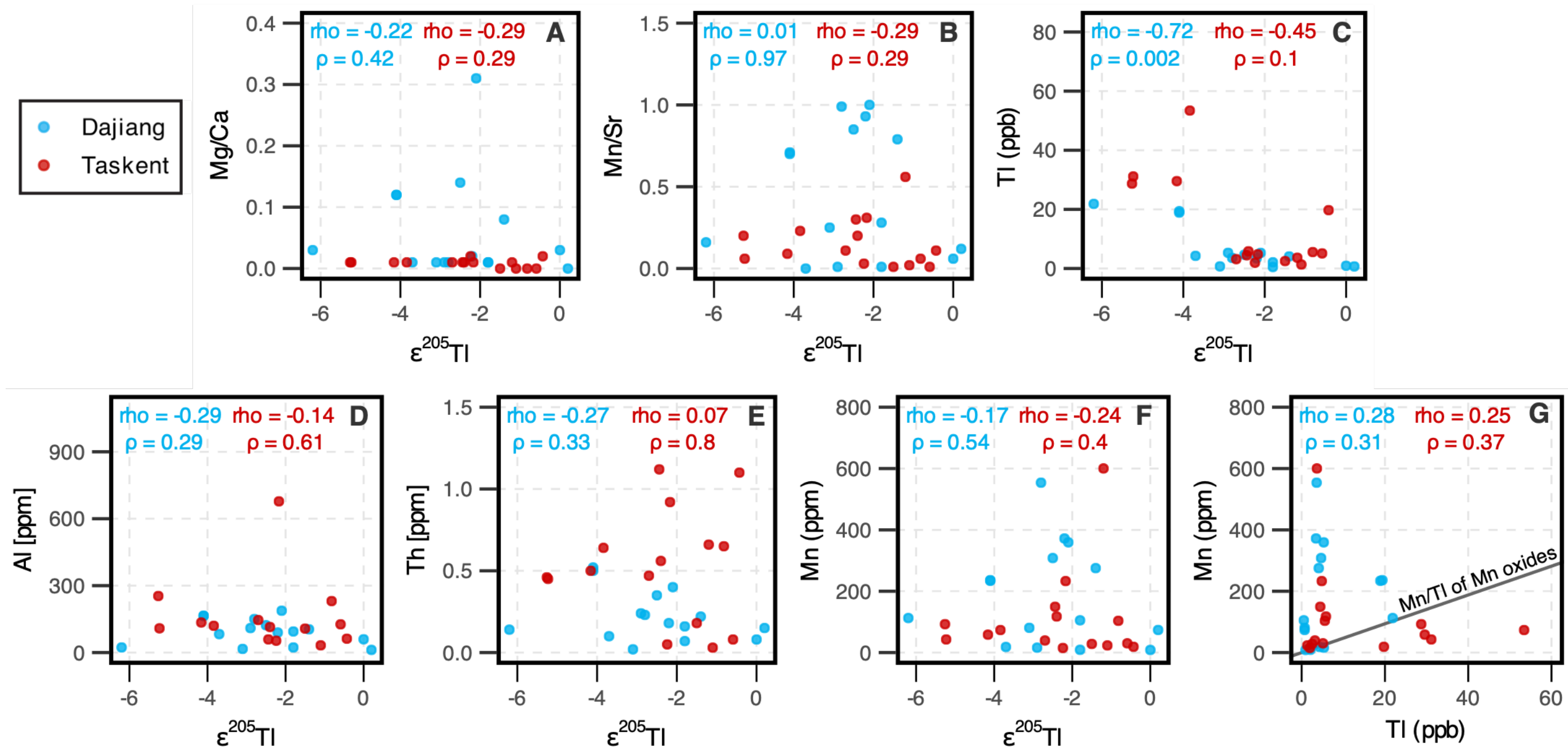

Fig. S1. Scatter plots of carbonate Tl isotope measurements and indicators of diagenesis and alteration.

$\varepsilon^{205}Tl_{carb}$ and **A**) Mg/Ca, **B**) Mn/Sr, **C**) Tl, **D**) Al, **E**) Th, and **F**) Mn concentrations, as well as **G**) Mn and Tl concentrations in the carbonate rock samples, plotted alongside the average Mn/Tl ratio in hydrogenetic ferromanganese crusts (dark grey line) (50). Rho and p-values are calculated independently for both the Dajiang and Taşkent sections (see *Statistical Analysis* for additional discussion of these tests).

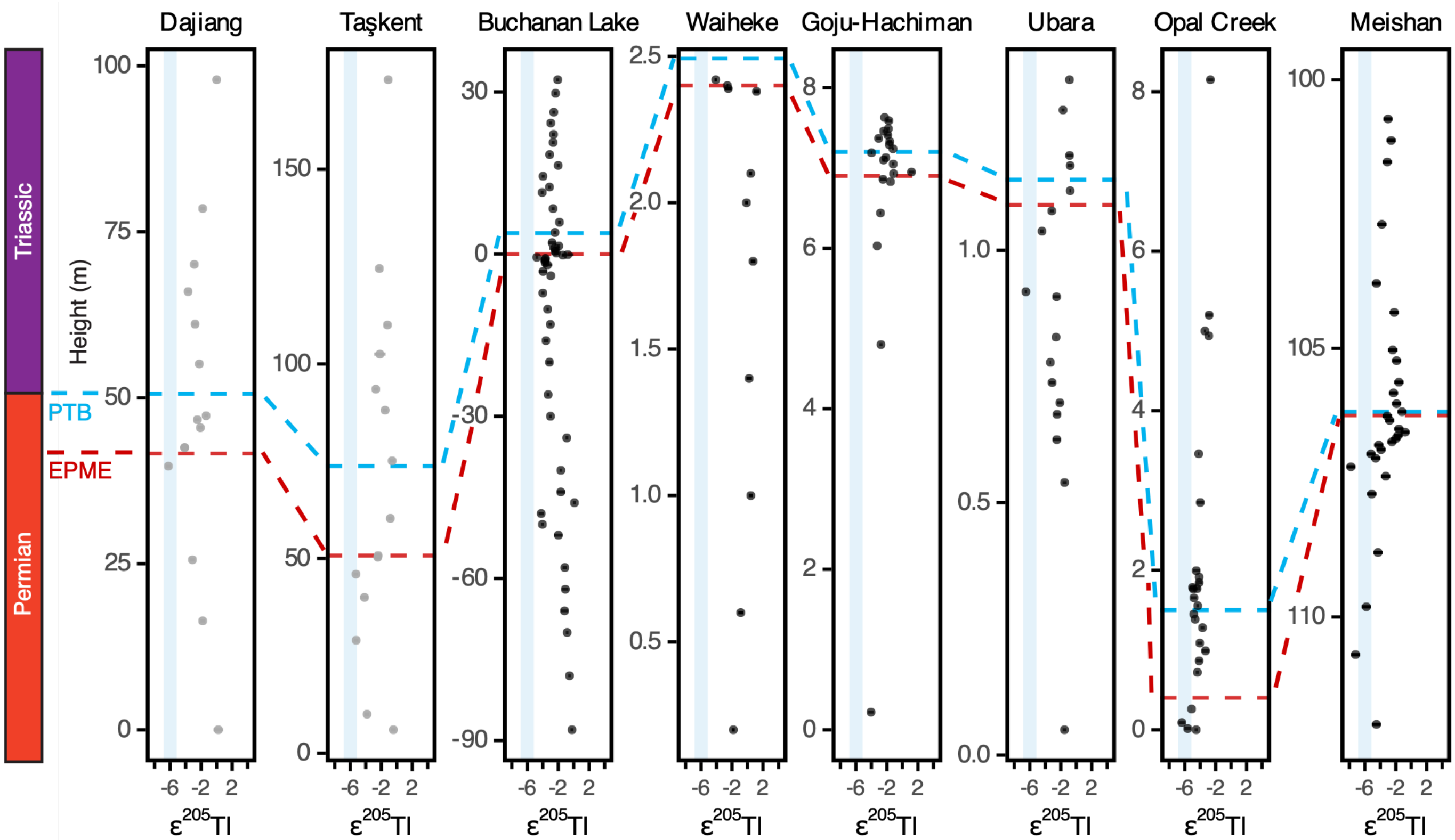

Fig. S2. Comparison of new and pre-existing ε^{205}Tl records.
Tl isotope data from Dajiang, Taşkent, Buchanan Lake, and Waiheke (this study) alongside published Permian-Triassic ε^{205}Tl records from the Goju-Hachiman, Ubara, and Opal Creek sections (18) and the Meishan section (60). The light blue lines represent the ε^{205}Tl of modern, well-oxygenated seawater, the red dashed line marks the end-Permian Mass Extinction (EPME; ca. 251.906 Ma), and the blue dashed line indicates the Permian-Triassic boundary (PTB; ca. 251.867 Ma). Error bars represent ±2 SD; where not visible, they are smaller than the plotted symbols.

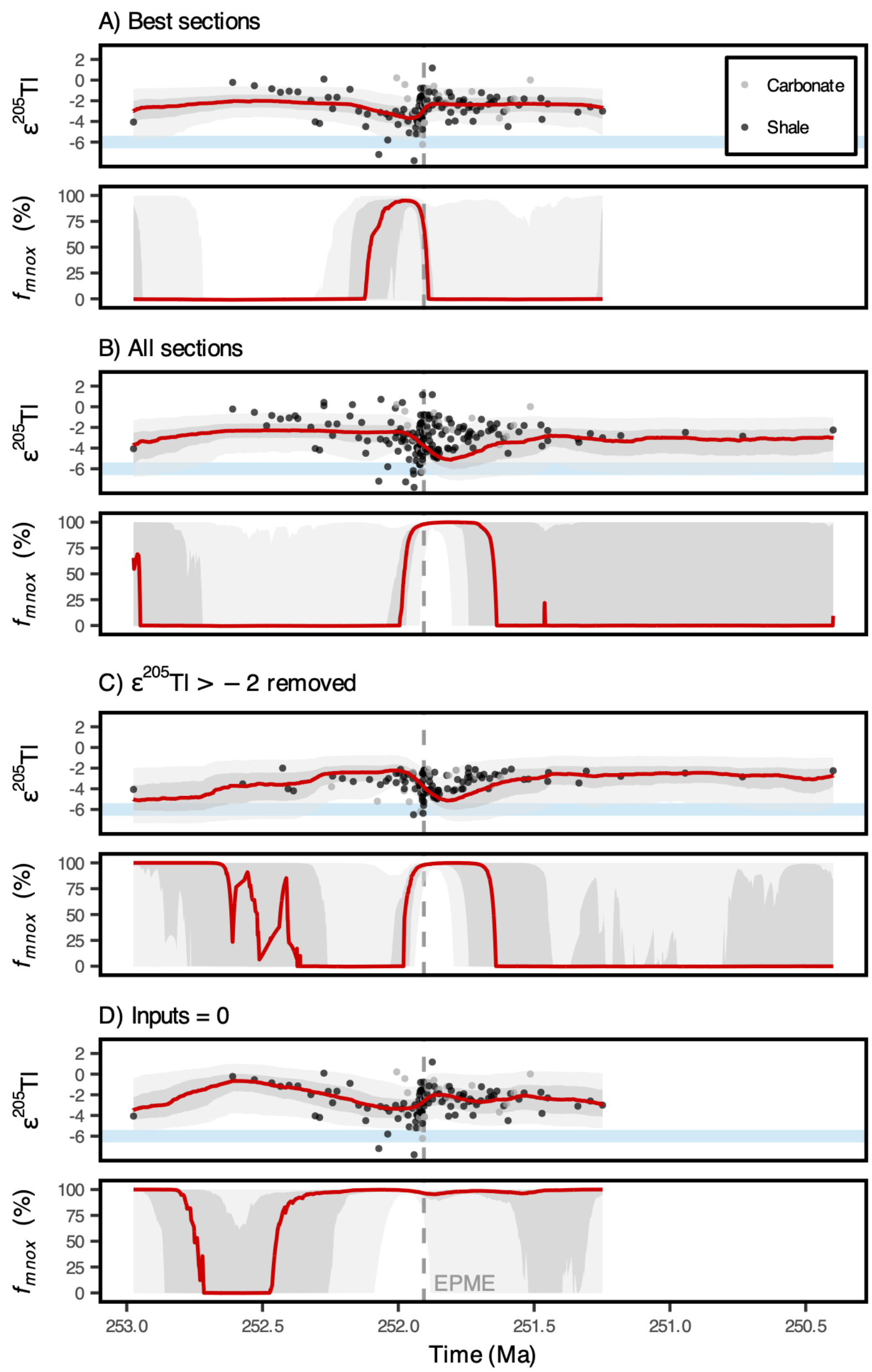

Fig. S3. Sensitivity analysis tests of the Tl inverse model.
Model results using **A**) data from only sections that extend both before and after the extinction and are likely unaltered (Dajiang, Taşkent, Buchanan Lake, Gujo-Hachiman, and Meishan), **B**) data from all eight sections, **C**) ε^{205}Tl data points higher than −2‱ removed, and **D**) using an ε^{205}Tl input value of 0‱ and data from the four sections that extend both before and after the extinction. Red lines represent the median model output, light grey shading indicates the 16th-84th percentile interval, and dark grey shading indicates the 33rd-66th percentile interval. The light blue line indicates the ε^{205}Tl values of modern, well-oxygenated seawater. The grey dashed line marks the end-Permian mass extinction.

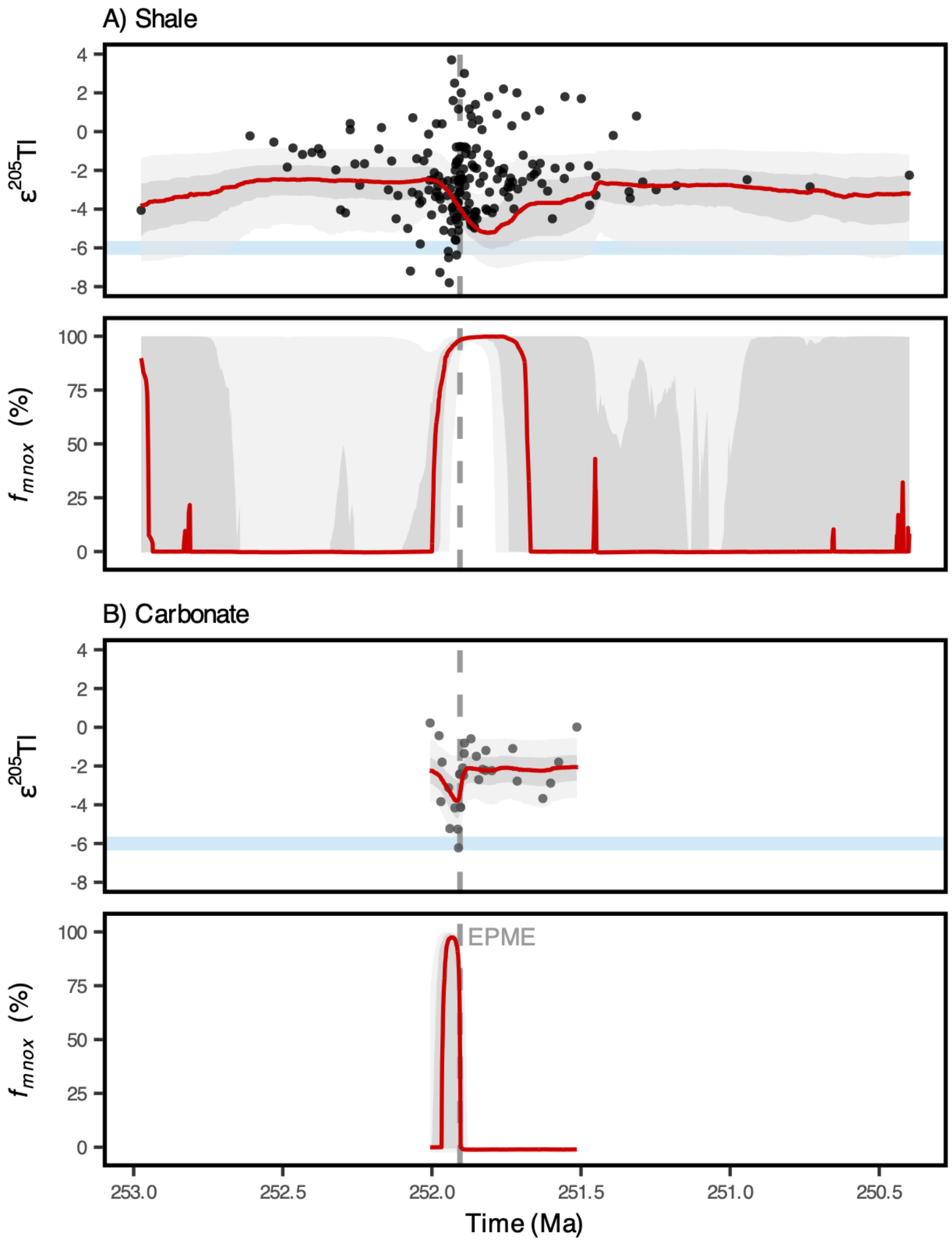

Fig. S4. Model sensitivity testing of carbonate versus shale lithologies.
A) Carbonate (Dajiang and Taşkent sections) and **B**) shale (Buchanan Lake, Waiheke, Gujo-Hachiman, Ubara, Opal Creek, and Meishan sections) modeling results. Red lines represent the median model output, light grey shading indicates the 16th-84th percentile interval, and dark grey shading indicates the 33rd-66th percentile interval. The light blue line indicates the ε^{205}Tl values of modern, well-oxygenated seawater. The grey dashed line marks the end-Permian mass extinction.

Table S1. Model parameters that inform the initial steady state calculation.

Parameter	Description	Steady-State Value	Units	Source
mols	Amount of Tl in the modern ocean	8.76×10^{10}	mols	(46)
J.in	Tl input flux	4.00×10^{6}	mols/yr	(115)
e205Tlin	Tl isotope value of input flux	-2	per mille	(47)
K.basalt	Rate constant of low-temperature basalt alteration output (65% of the inputs)	2.97×10^{-5}		Calculated (this study)
K.anox	Rate constant of anoxic sediments output (5% of the inputs)	1.10×10^{-3}		Calculated (this study)
K.mnoxide	Rate constant of Mn oxide burial output (30% of the inputs)	1.37×10^{-5}		Calculated (this study)
D.anox	Tl isotope fractionation associated with anoxic sediments	0	per mille	(47)
D.mnoxide	Tl isotope fractionation associated with Mn oxide burial	+16	per mille	(50)
D.basalt	Tl isotope fractionation associated with low-temperature basalt alteration	-1.2	per mille	(47)
f_anox	Modern fraction of seafloor anoxia	0.00211		(118)

Data S1. Geochemical data and age models.

Thallium isotope and trace metal data for Dajiang, Taşkent, Buchanan Lake, and Waiheke, as well as carbon and oxygen isotopes when available. This file also includes tie points used to develop the age models for these four sections and for the Ubara, Opal Creek, Gujo-Hachiman, and Meishan sections.

REFERENCES

1. X. Dai, M. Ovtcharova, J. H. F. L. Davies, H. Song, X. Liu, Z. Yuan, Y. Cao, R. Bai, D. Chu, J. Dal Corso, A. Brayard, S. Jiang, L. Miao, Y. Du, H. Song, Geochronology of the Early Triassic based on coupled Bayesian zircon eruption age and Bayesian age–depth models. *Proc. Natl. Acad. Sci. U.S.A.* **122**, e2509247122 (2025).

2. D. H. Erwin, *Extinction: How Life on Earth Nearly Ended 250 Million Years Ago* (Princeton Univ. Press, 2006).

3. H. Svensen, S. Planke, A. G. Polozov, N. Schmidbauer, F. Corfu, Y. Y. Podladchikov, B. Jamtveit, Siberian gas venting and the end-Permian environmental crisis. *Earth Planet. Sci. Lett.* **277**, 490–500 (2009).

4. J. Dal Corso, H. Song, S. Callegaro, D. Chu, Y. Sun, J. Hilton, S. E. Grasby, M. M. Joachimski, P. B. Wignall, Environmental crises at the Permian–Triassic mass extinction. *Nat. Rev. Earth Environ.* **3**, 197–214 (2022).

5. S. D. Burgess, J. D. Muirhead, S. A. Bowring, Initial pulse of Siberian Traps sills as the trigger of the end-Permian mass extinction. *Nat. Commun.* **8**, 164 (2017).

6. Q. Jiang, F. Jourdan, H. K. H. Olierook, R. E. Merle, J. Bourdet, D. Fougerouse, B. Godel, A. T. Walker, Volume and rate of volcanic CO_2 emissions governed the severity of past environmental crises. *Proc. Natl. Acad. Sci. U.S.A.* **119**, e2202039119 (2022).

7. S. D. Burgess, S. A. Bowring, High-precision geochronology confirms voluminous magmatism before, during, and after Earth's most severe extinction. *Sci. Adv.* **1**, e1500470 (2015).

8. Y. Sun, M. M. Joachimski, P. B. Wignall, C. Yan, Y. Chen, H. Jiang, L. Wang, X. Lai, Lethally hot temperatures during the Early Triassic greenhouse. *Science* **338**, 366–370 (2012).

9. P. B. Wignall, R. J. Twitchett, Oceanic anoxia and the end Permian mass extinction. *Science* **272**, 1155–1158 (1996).

10. J. L. Penn, C. Deutsch, J. L. Payne, E. A. Sperling, Temperature-dependent hypoxia explains biogeography and severity of end-Permian marine mass extinction. *Science* **362**, eaat1327 (2018).

11. K. Grice, C. Cao, G. D. Love, M. E. Böttcher, R. J. Twitchett, E. Grosjean, R. E. Summons, S. C. Turgeon, W. Dunning, Y. Jin, Photic zone euxinia during the Permian-Triassic superanoxic event. *Science* **307**, 706–709 (2005).

12. S. E. Grasby, B. Beauchamp, Latest Permian to Early Triassic basin-to-shelf anoxia in the Sverdrup Basin, Arctic Canada. *Chem. Geol.* **264**, 232–246 (2009).

13. D. P. G. Bond, P. B. Wignall, Pyrite framboid study of marine Permian–Triassic boundary sections: A complex anoxic event and its relationship to contemporaneous mass extinction. *GSA Bull.* **122**, 1265–1279 (2010).

14. G. A. Brennecka, A. D. Herrmann, T. J. Algeo, A. D. Anbar, Rapid expansion of oceanic anoxia immediately before the end-Permian mass extinction. *Proc. Natl. Acad. Sci. U.S.A.* **108**, 17631–17634 (2011).

15. K. V. Lau, K. Maher, D. Altiner, B. M. Kelley, L. R. Kump, D. J. Lehrmann, J. C. Silva-Tamayo, K. L. Weaver, M. Yu, J. L. Payne, Marine anoxia and delayed Earth system recovery after the end-Permian extinction. *Proc. Natl. Acad. Sci. U.S.A.* **113**, 2360–2365 (2016).

16. F. Zhang, S. Shen, Y. Cui, T. M. Lenton, T. W. Dahl, H. Zhang, Q. Zheng, W. Wang, K. Krainer, A. D. Anbar, Two distinct episodes of marine anoxia during the Permian-Triassic crisis evidenced by uranium isotopes in marine dolostones. *Geochim. Cosmochim. Acta* **287**, 165–179 (2020).

17. S. E. Grasby, D. P. G. Bond, P. B. Wignall, R. Yin, L. J. Strachan, S. Takahashi, Transient Permian-Triassic euxinia in the southern Panthalassa deep ocean. *Geology* **49**, 889–893 (2021).

18. S. M. Newby, J. D. Owens, S. D. Schoepfer, T. J. Algeo, Transient ocean oxygenation at end-Permian mass extinction onset shown by thallium isotopes. *Nat. Geosci.* **14**, 678–683 (2021).

19. S. Takahashi, R. S. Hori, S. Yamakita, Y. Aita, A. Takemura, M. Ikehara, Y. Xiong, S. W. Poulton, P. B. Wignall, T. Itai, H. J. Campbell, B. K. Spörli, Progressive development of ocean anoxia in the end-Permian pelagic Panthalassa. *Glob. Planet. Change* **207**, 103650 (2021).

20. Y. Zhang, H. Wen, C. Zhu, H. Fan, J. Xiao, J. Wen, Molybdenum isotopic evidence for anoxic marine conditions during the end-Permian mass extinction. *Chem. Geol.* **575**, 120259 (2021).

21. F. Zhang, S. J. Romaniello, T. J. Algeo, K. V. Lau, M. E. Clapham, S. Richoz, A. D. Herrmann, H. Smith, M. Horacek, A. D. Anbar, Multiple episodes of extensive marine anoxia linked to global warming and continental weathering following the latest Permian mass extinction. *Sci. Adv.* **4**, e1602921 (2018).

22. B. C. Proemse, S. E. Grasby, M. E. Wieser, B. Mayer, B. Beauchamp, Molybdenum isotopic evidence for oxic marine conditions during the latest Permian extinction. *Geology* **41**, 967–970 (2013).

23. M. Pimentel-Galvan, K. V. Lau, K. Maher, T. Mukerji, D. J. Lehrmann, D. Altiner, J. L. Payne, Duration and intensity of end-Permian marine anoxia. *Geochem. Geophys. Geosyst.* **23**, e2021GC010130 (2022).

24. M. A. Kipp, F. L. H. Tissot, Inverse methods for consistent quantification of seafloor anoxia using uranium isotope data from marine sediments. *Earth Planet. Sci. Lett.* **577**, 117240 (2022).

25. G. J. Gilleaudeau, X. Chen, S. J. Romaniello, S. A. Akam, C. Wittkop, S. Katsev, A. D. Anbar, E. D. Swanner, Uranium isotope systematics of a low-productivity ferruginous ocean analog: Implications for the uranium isotope record of early Earth. *Geochim. Cosmochim. Acta* **392**, 195–206 (2025).

26. B. Kendall, M. B. Andersen, J. D. Owens, "Assessing the effect of large igneous provinces on global oceanic redox conditions using non-traditional metal isotopes (molybdenum, uranium, thallium)" in *Large Igneous Provinces*, Geophysical Monograph Series, R. E. Ernst, A. J. Dickson, A. Bekker, Eds. (Wiley, ed. 1, 2021), pp. 305–323.

27. K. V. Lau, S. J. Romaniello, F. Zhang, *The Uranium Isotope Paleoredox Proxy*, Elements in Geochemical Tracers in Earth System Science (Cambridge Univ. Press, 2019).

28. S. R. Hlohowskyj, A. Chappaz, A. J. Dickson, *Molybdenum as a Paleoredox Proxy: Past, Present, and Future*, Elements in Geochemical Tracers in Earth System Science (Cambridge Univ. Press, 2021).

29. Y. Wang, K. M. Costa, W. Lu, S. K. V. Hines, S. G. Nielsen, Global oceanic oxygenation controlled by the Southern Ocean through the last deglaciation. *Sci. Adv.* **10**, eadk2506 (2024).

30. Z. Li, D. B. Cole, S. M. Newby, J. D. Owens, B. Kendall, C. T. Reinhard, New constraints on mid-Proterozoic ocean redox from stable thallium isotope systematics of black shales. *Geochim. Cosmochim. Acta* **315**, 185–206 (2021).

31. Y. Kakuwa, R. Matsumoto, Cerium negative anomaly just before the Permian and Triassic boundary event—The upward expansion of anoxia in the water column. *Palaeogeogr. Palaeoclimatol. Palaeoecol.* **229**, 335–344 (2006).

32. C. M. Ostrander, J. D. Owens, S. G. Nielsen, Constraining the rate of oceanic deoxygenation leading up to a Cretaceous Oceanic Anoxic Event (OAE-2: ~94 Ma). *Sci. Adv.* **3**, e1701020 (2017).

33. C. M. Ostrander, S. G. Nielsen, J. D. Owens, B. Kendall, G. W. Gordon, S. J. Romaniello, A. D. Anbar, Fully oxygenated water columns over continental shelves before the Great Oxidation Event. *Nat. Geosci.* **12**, 186–191 (2019).

34. C. M. Ostrander, J. N. R. Clemente, R. G. Stockey, J. V. Strauss, T. Fraser, S. G. Nielsen, E. A. Sperling, Dynamic deep marine oxygenation during the Early and Middle Paleozoic. *Sci. Adv.* **11**, eadw5878 (2025).

35. T. R. Them, B. C. Gill, A. H. Caruthers, A. M. Gerhardt, D. R. Gröcke, T. W. Lyons, S. M. Marroquín, S. G. Nielsen, J. P. Trabucho Alexandre, J. D. Owens, Thallium isotopes reveal protracted anoxia during the Toarcian (Early Jurassic) associated with volcanism, carbon burial, and mass extinction. *Proc. Natl. Acad. Sci. U.S.A.* **115**, 6596–6601 (2018).

36. J. N. R. Clemente, H. Fan, C. M. Ostrander, H. Zhang, H. Wen, E. A. Sperling, S. G. Nielsen, Thallium isotopes suggest the global deep ocean did not approach modern oxygenation during Cambrian age 3 Metazoan radiation. *Geobiology* **23**, e70028 (2025).

37. L. Wang, M. Cao, Y. Lin, F. Wu, Q. Tang, F. Zhang, Reconstruction of marine redox landscape during the Cryogenian interglacial oceans using thallium isotopes. *Earth Planet. Sci. Lett.* **662**, 119419 (2025).

38. C. M. Ostrander, J. D. Owens, S. G. Nielsen, T. W. Lyons, Y. Shu, X. Chen, E. A. Sperling, G. Jiang, D. T. Johnston, S. K. Sahoo, A. D. Anbar, Thallium isotope ratios in shales from South China and northwestern Canada suggest widespread O_2 accumulation in marine bottom waters was an uncommon occurrence during the Ediacaran Period. *Chem. Geol.* **557**, 119856 (2020).

39. C. M. Ostrander, C. J. Bjerrum, A.-S. C. Ahm, S. R. Stenger, K. D. Bergmann, M. A. K. El-Ghali, A. R. Harthi, Z. Aisri, S. G. Nielsen, Widespread seafloor anoxia during generation of the Ediacaran Shuram carbon isotope excursion. *Geobiology* **21**, 556–570 (2023).

40. S. G. Nielsen, M. Goff, S. P. Hesselbo, H. C. Jenkyns, D. E. LaRowe, C.-T. A. Lee, Thallium isotopes in early diagenetic pyrite—A paleoredox proxy? *Geochim. Cosmochim. Acta* **75**, 6690–6704 (2011).

41. J. D. Owens, S. G. Nielsen, T. J. Horner, C. M. Ostrander, L. C. Peterson, Thallium-isotopic compositions of euxinic sediments as a proxy for global manganese-oxide burial. *Geochim. Cosmochim. Acta* **213**, 291–307 (2017).

42. D. B. Kemp, P. M. Sadler, Climatic and eustatic signals in a global compilation of shallow marine carbonate accumulation rates. *Sedimentology* **61**, 1286–1297 (2014).

43. P. M. Sadler, The expected duration of upward-shallowing peritidal carbonate cycles and their terminal hiatuses. *GSA Bull.* **106**, 791–802 (1994).

44. Y. Shu, C. M. Ostrander, D. S. Hardisty, P. K. Swart, S. G. Nielsen, Extending the utility of the Tl isotope paleoredox proxy to carbonates. *Geochim. Cosmochim. Acta* **394**, 32–42 (2025).

45. M. Rehkämper, M. Frank, J. R. Hein, A. Halliday, Cenozoic marine geochemistry of thallium deduced from isotopic studies of ferromanganese crusts and pelagic sediments. *Earth Planet. Sci. Lett.* **219**, 77–91 (2004).

46. M. Rehkämper, S. G. Nielsen, The mass balance of dissolved thallium in the oceans. *Mar. Chem.* **85**, 125–139 (2004).

47. S. G. Nielsen, M. Rehkämper, J. Prytulak, Investigation and application of thallium isotope fractionation. *Rev. Mineral. Geochem.* **82**, 759–798 (2017).

48. S. G. Nielsen, M. Rehkämper, D. Porcelli, P. Andersson, A. N. Halliday, P. W. Swarzenski, C. Latkoczy, D. Günther, Thallium isotope composition of the upper continental crust and rivers—An investigation of the continental sources of dissolved marine thallium. *Geochim. Cosmochim. Acta* **69**, 2007–2019 (2005).

49. Y. Wang, W. Lu, K. M. Costa, S. G. Nielsen, Beyond anoxia: Exploring sedimentary thallium isotopes in paleo-redox reconstructions from a new core top collection. *Geochim. Cosmochim. Acta* **333**, 347–361 (2022).

50. M. Rehkämper, M. Frank, J. R. Hein, D. Porcelli, A. Halliday, J. Ingri, V. Liebetrau, Thallium isotope variations in seawater and hydrogenetic, diagenetic, and hydrothermal ferromanganese deposits. *Earth Planet. Sci. Lett.* **197**, 65–81 (2002).

51. C. L. Peacock, E. M. Moon, Oxidative scavenging of thallium by birnessite: Explanation for thallium enrichment and stable isotope fractionation in marine ferromanganese precipitates. *Geochim. Cosmochim. Acta* **84**, 297–313 (2012).

52. S. G. Nielsen, L. E. Wasylenki, M. Rehkämper, C. L. Peacock, Z. Xue, E. M. Moon, Towards an understanding of thallium isotope fractionation during adsorption to manganese oxides. *Geochim. Cosmochim. Acta* **117**, 252–265 (2013).

53. R. F. Phillips, Y. Wang, F. Klein, G. Farfan, C. M. Ostrander, H. Gadol, C. M. Hansel, S. G. Nielsen, The role of manganese oxide mineralogy in thallium isotopic fractionation upon sorption. *Geochim. Cosmochim. Acta* **356**, 83–92 (2023).

54. B. G. Clement, G. W. Luther III, B. M. Tebo, Rapid, oxygen-dependent microbial Mn(II) oxidation kinetics at sub-micromolar oxygen concentrations in the Black Sea suboxic zone. *Geochim. Cosmochim. Acta* **73**, 1878–1889 (2009).

55. G. J. Dick, B. G. Clement, S. M. Webb, F. J. Fodrie, J. R. Bargar, B. M. Tebo, Enzymatic microbial Mn(II) oxidation and Mn biooxide production in the Guaymas Basin deep-sea hydrothermal plume. *Geochim. Cosmochim. Acta* **73**, 6517–6530 (2009).

56. S. E. Calvert, T. F. Pedersen, Sedimentary geochemistry of manganese; implications for the environment of formation of manganiferous black shales. *Econ. Geol.* **91**, 36–47 (1996).

57. U. Brand, J. Veizer, Chemical diagenesis of a multicomponent carbonate system; 1, Trace elements. *J. Sediment. Res.* **50**, 1219–1236 (1980).

58. J. Knies, S. E. Grasby, B. Beauchamp, C. J. Schubert, Water mass denitrification during the latest Permian extinction in the Sverdrup Basin, Arctic Canada. *Geology* **41**, 167–170 (2013).

59. R. V. Tyson, T. H. Pearson, Modern and ancient continental shelf anoxia: An overview. *Geol. Soc. Lond. Spec. Publ.* **58**, 1–24 (1991).

60. L. Wang, M. Cao, Y.-B. Lin, F. Wu, Y. Wang, H. Zhang, S.-Z. Shen, F. Zhang, Thallium isotopes constrain the timing and tempo of oceanic deoxygenation across the end-Permian mass extinction. *Earth Planet. Sci. Lett.* **681**, 119937 (2026).

61. J. Wang, L. Shao, X. Wang, The coal-forming environment at the end of the Late Permian and its control on trace elements: The Upper Xuanwei Formation in eastern Yunnan, China. *Processes* **11**, 2936 (2023).

62. S. E. Grasby, B. Beauchamp, Intrabasin variability of the carbon-isotope record across the Permian–Triassic transition, Sverdrup Basin, Arctic Canada. *Chem. Geol.* **253**, 141–150 (2008).

63. K. M. Meyer, M. Yu, A. B. Jost, B. M. Kelley, J. L. Payne, $\delta^{13}C$ evidence that high primary productivity delayed recovery from end-Permian mass extinction. *Earth Planet. Sci. Lett.* **302**, 378–384 (2011).

64. A. F. Embry, "Mesozoic history of the Arctic Islands" in *Geology of the Innuitian Orogen and Arctic Platform of Canada and Greenland*, H. P. Trettin, Ed. (Geological Society of America, 1991), vol. 3.

65. D. J. Lehrmann, W. Jiayong, P. Enos, Controls on facies architecture of a large Triassic carbonate platform; the Great Bank of Guizhou, Nanpanjiang Basin, South China. *J. Sediment. Res.* **68**, 311–326 (1998).

66. J. R. Groves, D. Altiner, R. Rettori, Extinction, survival, and recovery of lagenide foraminifers in the Permian–Triassic boundary interval, central Taurides, Turkey. *J. Paleontol.* **79**, 1–38 (2005).

67. R. S. Hori, S. Yamakita, M. Ikehara, K. Kodama, Y. Aita, T. Sakai, A. Takemura, Y. Kamata, N. Suzuki, S. Takahashi, K. B. Spörli, J. A. Grant-Mackie, Early Triassic (Induan) Radiolaria and carbon-isotope ratios of a deep-sea sequence from Waiheke Island, North Island, New Zealand. *Palaeoworld* **20**, 166–178 (2011).

68. B. Beauchamp, J. C. Harrison, J. Utting, T. A. Brent, S. Pinard, "Carboniferous and Permian Subsurface Stratigraphy, Prince Patrick Island, Northwest Territories, Canadian Arctic" (Natural Resources Canada, 2001); https://ostrnrcan-dostrncan.canada.ca/handle/1845/185734.

69. S. Howarth, J. Prytulak, S. H. Little, S. J. Hammond, M. Widdowson, Thallium concentration and thallium isotope composition of lateritic terrains. *Geochim. Cosmochim. Acta* **239**, 446–462 (2018).

70. W. He, G. R. Shi, K. Zhang, N. Suzuki, H. Wang, F. Yang, Y. Xiao, T. Yang, Y. Huang, C. Wu, K. Jiang, The deterioration and collapse of late Permian marine ecosystems and the end-Permian mass extinction: A global view. *Earth Sci. Rev.* **261**, 104971 (2025).

71. Y. Isozaki, Permo-Triassic boundary superanoxia and stratified superocean: Records from lost deep sea. *Science* **276**, 235–238 (1997).

72. F. Zhang, T. J. Algeo, S. J. Romaniello, Y. Cui, L. Zhao, Z.-Q. Chen, A. D. Anbar, Congruent Permian-Triassic δ^{238}U records at Panthalassic and Tethyan sites: Confirmation of global-oceanic anoxia and validation of the U-isotope paleoredox proxy. *Geology* **46**, 327–330 (2018).

73. M. Elrick, V. Polyak, T. J. Algeo, S. Romaniello, Y. Asmerom, A. D. Herrmann, A. Anbar, L. Zhao, Z. Q. Chen, Global-ocean redox variation during the middle-late Permian through Early Triassic based on uranium isotope and Th/U trends of marine carbonates. *Geology* **45**, 163–166 (2017).

74. M. B. Andersen, S. Romaniello, D. Vance, S. H. Little, R. Herdman, T. W. Lyons, A modern framework for the interpretation of ^{238}U/^{235}U in studies of ancient ocean redox. *Earth Planet. Sci. Lett.* **400**, 184–194 (2014).

75. F. Qian, Y. Wang, K. M. Costa, S. G. Nielsen, Sedimentary thallium isotopes as a proxy for reconstructing global oceanic oxygenation during millennial-scale events. *Geochim. Cosmochim. Acta* **416**, 319–333 (2026).

76. M. van Hulten, R. Middag, J.-C. Dutay, H. de Baar, M. Roy-Barman, M. Gehlen, A. Tagliabue, A. Sterl, Manganese in the west Atlantic Ocean in the context of the first global ocean circulation model of manganese. *Biogeosciences* **14**, 1123–1152 (2017).

77. D. Hülse, K. V. Lau, S. J. van de Velde, S. Arndt, K. M. Meyer, A. Ridgwell, End-Permian marine extinction due to temperature-driven nutrient recycling and euxinia. *Nat. Geosci.* **14**, 862–867 (2021).

78. D. H. Erwin, The end and the beginning: Recoveries from mass extinctions. *Trends Ecol. Evol.* **13**, 344–349 (1998).

79. S. Sahney, M. J. Benton, Recovery from the most profound mass extinction of all time. *Proc. R. Soc. London Ser. B* **275**, 759–765 (2008).

80. M. S. Fantle, B. D. Barnes, K. V. Lau, The role of diagenesis in shaping the geochemistry of the marine carbonate record. *Annu. Rev. Earth Planet. Sci.* **48**, 549–583 (2020).

81. H. A. Tourtelot, Black shale; its deposition and diagenesis. *Clays Clay Miner.* **27**, 313–321 (1979).

82. C. Montoya-Pino, S. Weyer, A. D. Anbar, J. Pross, W. Oschmann, B. van de Schootbrugge, H. W. Arz, Global enhancement of ocean anoxia during Oceanic Anoxic Event 2: A quantitative approach using U isotopes. *Geology* **38**, 315–318 (2010).

83. D. Asael, F. L. H. Tissot, C. T. Reinhard, O. Rouxel, N. Dauphas, T. W. Lyons, E. Ponzevera, C. Liorzou, S. Chéron, Coupled molybdenum, iron and uranium stable isotopes as oceanic paleoredox proxies during the Paleoproterozoic Shunga Event. *Chem. Geol.* **362**, 193–210 (2013).

84. S. K. Gangl, C. H. Stirling, H. C. Jenkyns, W. J. Preston, M. O. Clarkson, C. M. Moy, A. J. Dickson, D. Porcelli, Regional conditions cause contrasting behaviour in U-isotope fractionation in black shales: Constraints for global ocean palaeo-redox reconstructions. *Chem. Geol.* **623**, 121411 (2023).

85. A. R. Voegelin, T. F. Nägler, E. Samankassou, I. M. Villa, Molybdenum isotopic composition of modern and Carboniferous carbonates. *Chem. Geol.* **265**, 488–498 (2009).

86. S. J. Romaniello, A. D. Herrmann, A. D. Anbar, Syndepositional diagenetic control of molybdenum isotope variations in carbonate sediments from the Bahamas. *Chem. Geol.* **438**, 84–90 (2016).

87. Y. Kato, K. Nakao, Y. Isozaki, Geochemistry of Late Permian to Early Triassic pelagic cherts from southwest Japan: Implications for an oceanic redox change. *Chem. Geol.* **182**, 15–34 (2002).

88. J.-X. Fan, S.-Z. Shen, D. H. Erwin, P. M. Sadler, N. MacLeod, Q.-M. Cheng, X.-D. Hou, J. Yang, X.-D. Wang, Y. Wang, H. Zhang, X. Chen, G.-X. Li, Y.-C. Zhang, Y.-K. Shi, D.-X. Yuan, Q. Chen, L.-N. Zhang, C. Li, Y.-Y. Zhao, A high-resolution summary of Cambrian to Early Triassic marine invertebrate biodiversity. *Science* **367**, 272–277 (2020).

89. S. D. Burgess, S. Bowring, S.-Z. Shen, High-precision timeline for Earth's most severe extinction. *Proc. Natl. Acad. Sci. U.S.A.* **111**, 3316–3321 (2014).

90. T. J. Algeo, K. Kuwahara, H. Sano, S. Bates, T. Lyons, E. Elswick, L. Hinnov, B. Ellwood, J. Moser, J. B. Maynard, Spatial variation in sediment fluxes, redox conditions, and productivity in the Permian–Triassic Panthalassic Ocean. *Palaeogeogr. Palaeoclimatol. Palaeoecol.* **308**, 65–83 (2011).

91. S. D. Schoepfer, C. M. Henderson, G. H. Garrison, J. Foriel, P. D. Ward, D. Selby, J. C. Hower, T. J. Algeo, Y. Shen, Termination of a continent-margin upwelling system at the Permian–Triassic boundary (Opal Creek, Alberta, Canada). *Glob. Planet. Change* **105**, 21–35 (2013).

92. S.-Z. Shen, J. L. Crowley, Y. Wang, S. A. Bowring, D. H. Erwin, P. M. Sadler, C.-Q. Cao, D. H. Rothman, C. M. Henderson, J. Ramezani, H. Zhang, Y. Shen, X.-D. Wang, W. Wang, L. Mu, W.-Z. Li, Y.-G. Tang, X.-L. Liu, L.-J. Liu, Y. Zeng, Y.-F. Jiang, Y.-G. Jin, Calibrating the end-Permian mass extinction. *Science* **334**, 1367–1372 (2011).

93. J. Chen, G. Sun, B. Lu, R. Ma, Z. Xiao, Y.-F. Cai, H. Zhang, S.-Z. Shen, F. Zhang, Z. Feng, Inconsistent mercury records from terrestrial upland to coastal lowland across the Permian-Triassic transition. *Earth Planet. Sci. Lett.* **614**, 118195 (2023).

94. S. G. Nielsen, M. Rehkämper, J. Baker, A. N. Halliday, The precise and accurate determination of thallium isotope compositions and concentrations for water samples by MC-ICPMS. *Chem. Geol.* **204**, 109–124 (2004).

95. K. V. Lau, K. Maher, S. T. Brown, A. B. Jost, D. Altıner, D. J. DePaolo, A. Eisenhauer, B. M. Kelley, D. J. Lehrmann, A. Paytan, M. Yu, J. C. Silva-Tamayo, J. L. Payne, The influence of seawater carbonate chemistry, mineralogy, and diagenesis on calcium isotope variations in Lower-Middle Triassic carbonate rocks. *Chem. Geol.* **471**, 13–37 (2017).

96. E. S. Krull, D. J. Lehrmann, D. Druke, B. Kessel, Y. Yu, R. Li, Stable carbon isotope stratigraphy across the Permian–Triassic boundary in shallow marine carbonate platforms, Nanpanjiang Basin, south China. *Palaeogeogr. Palaeoclimatol. Palaeoecol.* **204**, 297–315 (2004).

97. D. J. Lehrmann, L. Stepchinski, D. Altiner, M. J. Orchard, P. Montgomery, P. Enos, B. B. Ellwood, S. A. Bowring, J. Ramezani, H. Wang, J. Wei, M. Yu, J. D. Griffiths, M. Minzoni, E. K. Schaal, X. Li, K. M. Meyer, J. L. Payne, An integrated biostratigraphy (conodonts and foraminifers) and chronostratigraphy (paleomagnetic reversals, magnetic susceptibility, elemental chemistry, carbon isotopes and geochronology) for the Permian–Upper Triassic strata of Guandao section, Nanpanjiang Basin, south China. *J. Asian Earth Sci.* **108**, 117–135 (2015).

98. D. Altıner, J. L. Payne, D. J. Lehrmann, S. Özkan-Altıner, B. M. Kelley, M. M. Summers, M. Yu, Triassic Foraminifera from the Great Bank of Guizhou, Nanpanjiang Basin, south China: Taxonomic account, biostratigraphy, and implications for recovery from end-Permian mass extinction. *J. Paleontol.* **95**, 1–53 (2021).

99. J. L. Payne, D. J. Lehrmann, J. Wei, M. J. Orchard, D. P. Schrag, A. H. Knoll, Large perturbations of the carbon cycle during recovery from the end-Permian extinction. *Science* **305**, 506–509 (2004).

100. J. L. Payne, A. V. Turchyn, A. Paytan, D. J. DePaolo, D. J. Lehrmann, M. Yu, J. Wei, Calcium isotope constraints on the end-Permian mass extinction. *Proc. Natl. Acad. Sci. U.S.A.* **107**, 8543–8548 (2010).

101. K. M. Meyer, M. Yu, D. Lehrmann, B. van de Schootbrugge, J. L. Payne, Constraints on Early Triassic carbon cycle dynamics from paired organic and inorganic carbon isotope records. *Earth Planet. Sci. Lett.* **361**, 429–435 (2013).

102. H. Song, J. Tong, T. J. Algeo, M. Horacek, H. Qiu, H. Song, L. Tian, Z.-Q. Chen, Large vertical $\delta^{13}C_{DIC}$ gradients in Early Triassic seas of the South China craton: Implications for oceanographic changes related to Siberian Traps volcanism. *Glob. Planet. Change* **105**, 7–20 (2013).

103. H. Song, J. Tong, T. J. Algeo, H. Song, H. Qiu, Y. Zhu, L. Tian, S. Bates, T. W. Lyons, G. Luo, L. R. Kump, Early Triassic seawater sulfate drawdown. *Geochim. Cosmochim. Acta* **128**, 95–113 (2014).

104. J. Wang, A. D. Jacobson, H. Zhang, J. Ramezani, B. B. Sageman, M. T. Hurtgen, S. A. Bowring, S.-Z. Shen, Coupled $\delta^{44/40}$Ca, $\delta^{88/86}$Sr, and ^{87}Sr/^{86}Sr geochemistry across the end-Permian mass extinction event. *Geochim. Cosmochim. Acta* **262**, 143–165 (2019).

105. J. L. Payne, D. J. Lehrmann, J. Wei, A. H. Knoll, The pattern and timing of biotic recovery from the end-Permian extinction on the Great Bank of Guizhou, Guizhou Province, China. *PALAIOS* **21**, 63–85 (2006).

106. J. L. Payne, M. Summers, B. L. Rego, D. Altiner, J. Wei, M. Yu, D. J. Lehrmann, Early and Middle Triassic trends in diversity, evenness, and size of foraminifers on a carbonate platform in south China: Implications for tempo and mode of biotic recovery from the end-Permian mass extinction. *Paleobiology* **37**, 409–425 (2011).

107. H. Song, P. B. Wignall, Z.-Q. Chen, J. Tong, D. P. G. Bond, X. Lai, X. Zhao, H. Jiang, C. Yan, Z. Niu, J. Chen, H. Yang, Y. Wang, Recovery tempo and pattern of marine ecosystems after the end-Permian mass extinction. *Geology* **39**, 739–742 (2011).

108. H. Song, P. B. Wignall, J. Tong, H. Yin, Two pulses of extinction during the Permian–Triassic crisis. *Nat. Geosci.* **6**, 52–56 (2013).

109. H. Sanei, S. E. Grasby, B. Beauchamp, Latest Permian mercury anomalies. *Geology* **40**, 63–66 (2012).

110. S. E. Grasby, W. Shen, R. Yin, J. D. Gleason, J. D. Blum, R. F. Lepak, J. P. Hurley, B. Beauchamp, Isotopic signatures of mercury contamination in latest Permian oceans. *Geology* **45**, 55–58 (2017).

111. M. Li, S. E. Grasby, S.-J. Wang, X. Zhang, L. E. Wasylenki, Y. Xu, M. Sun, B. Beauchamp, D. Hu, Y. Shen, Nickel isotopes link Siberian Traps aerosol particles to the end-Permian mass extinction. *Nat. Commun.* **12**, 2024 (2021).

112. K. Kodama, M. Fukuoka, Y. Aita, T. Sakai, R.S. Hori., A Takemura, H.J. Campbell, C.J. Hollis, J.A. Grant-Mackie, K.B. Sporli, "Paleomagnetic results from arrow rocks in the framework of paleomagnetism in pre-Neogene rocks from New Zealand" in *The Oceanic Permian/Triassic Boundary Sequence at Arrow Rocks (Oruatemanu), Northland, New Zealand*, GNS Science Monograph, (GNS Science, 2007) vol. 24.

113. S. Takahashi, K. Kaiho, R. S. Hori, P. Gorjan, T. Watanabe, S. Yamakita, Y. Aita, A. Takemura, K. B. Spörli, T. Kakegawa, M. Oba, Sulfur isotope profiles in the pelagic Panthalassic deep sea during the Permian–Triassic transition. *Glob. Planet. Change* **105**, 68–78 (2013).

114. C. M. Ostrander, S. G. Nielsen, H. J. Gadol, L. Villarroel, S. D. Wankel, T. J. Horner, J. Blusztajn, C. M. Hansel, Thallium isotope cycling between waters, particles, and sediments across a redox gradient. *Geochim. Cosmochim. Acta* **348**, 397–409 (2023).

115. J. D. Owens, *Application of Thallium Isotopes: Tracking Marine Oxygenation through Manganese Oxide Burial* (Cambridge Univ. Press, ed. 1, 2019).

116. N. Metropolis, A. W. Rosenbluth, M. N. Rosenbluth, A. H. Teller, E. Teller, Equation of state calculations by fast computing machines. *J. Chem. Phys.* **21**, 1087–1092 (1953).

117. W. K. Hastings, Monte Carlo sampling methods using Markov chains and their applications. *Biometrika* **57**, 97–109 (1970).

118. J. J. Helly, L. A. Levin, Global distribution of naturally occurring marine hypoxia on continental margins. *Deep Sea Res. I Oceanogr. Res. Pap.* **51**, 1159–1168 (2004).
